# Comparative transcriptomic analyses of diploid and tetraploid citrus reveal how ploidy level influences salt stress tolerance

**DOI:** 10.3389/fpls.2024.1469115

**Published:** 2024-10-30

**Authors:** Marie Bonnin, Alexandre Soriano, Bénédicte Favreau, Radia Lourkisti, Maëva Miranda, Patrick Ollitrault, Julie Oustric, Liliane Berti, Jérémie Santini, Raphaël Morillon

**Affiliations:** ^1^ Projet Ressources Naturelles Axe Adaptation des végé taux aux changements globaux, Unité Mixte de Recherche Centre National de la Recherche Scientifique (UMR CNRS) 6134 Science Pour l’Environment (SPE), Universitéde Corse, Corsica, France; ^2^ Unité Mixte de Recherche Amélioration Génétique et Adaptation des Plantes méditerranéennes et tropicales (UMR AGAP) Institut, Univ. Montpellier, Centre de coopération Internationale en Recherche Agronomique pour le Développement (CIRAD), Institut National de Recherche pour l’Agriculture, l’Alimentation et l’Environnement (INRAE), Institut Agro, Montpellier, France

**Keywords:** citrus, differentially expressed genes, salt stress, tetraploid, transcriptomic study

## Abstract

**Introduction:**

Citrus is an important fruit crop for human health. The sensitivity of citrus trees to a wide range of abiotic stresses is a major challenge for their overall growth and productivity. Among these abiotic stresses, salinity results in a significant loss of global citrus yield. In order to find straightforward and sustainable solutions for the future and to ensure citrus productivity, it is of paramount importance to decipher the mechanisms responsible for salinity stress tolerance. Thisstudy aimed to investigate how ploidy levels influence salt stress tolerance in citrus by comparing the transcriptomic responses of diploid and tetraploid genotypes. In a previous article we investigated the physiological and biochemical response of four genotypes with different ploidy levels: diploid trifoliate orange (Poncirus trifoliata [L.] Raf.) (PO2x) and Cleopatra mandarin (Citrus reshni Hort. Ex Tan.) (CL2x) and their respective tetraploids (PO4x, CL4x).

**Methods:**

In this study, we useda multifactorial gene selection and gene clustering approach to finely dissect the influence of ploidy level on the salt stress response of each genotype. Following transcriptome sequencing, differentially expressed genes (DEGs) were identified in response to salt stress in leaves and roots of the different citrus genotypes.

**Result and discussion:**

Gene expression profiles and functional characterization of genes involved in the response to salt stress, as a function of ploidy level and the interaction between stress response and ploidy level, have enabled us to highlight the mechanisms involved in the varieties tested. Saltstress induced overexpression of carbohydrate biosynthesis and cell wall remodelling- related genes specifically in CL4x Ploidy level enhanced oxidative stress response in PO and ion management capacity in both genotypes. Results further highlighted that under stress conditions, only the CL4x genotype up- regulated genes involved in sugar biosynthesis, transport management, cell wall remodelling, hormone signalling, enzyme regulation and antioxidant metabolism. These findings provide crucial insights that could inform breeding strategies for developing salt-tolerant citrus varieties.

## 1 Introduction

Citrus fruits are among the most important fruit crops worldwide. In the Mediterranean Basin and several other countries, they are mainly cultivated in coastal areas (Spreen et al., 2020), where they are increasingly affected by abiotic stress such as drought, extreme temperature and salinity (Colmenero-Flores et al., 2020). Salinity can reduce growth in citrus trees and cause physiological disorders (Bartels and Sunkar, 2005; Aouad et al., 2015; Ben Yahmed et al., 2015). Citrus crops are highly salt sensitive, and their response to soil salinity depends on the rootstock and scion, the soil type, the irrigation system that is used and the climate (Sykes, 2011; Ziogas et al., 2021). Salinity damages happen when the dissolved salt in water reduces the amount of water available in the root zone for hydration (Benyahia and Aditi, 2022). Movement of water in the leaf tissues of citrus can cause accumulation of chloride (Cl^-^) ions that in turn affect the transpiration and photosynthesis (Allario et al., 2011; Ashraf and Harris, 2013). Increasing Cl^-^ concentration accelerates defoliation by enhancing leaf abscission and ethylene production (Brumós et al., 2009; Hussain et al., 2012). Our previous work demonstrated that tetraploidy enhances photosynthetic and antioxidant capacities under salt stress. Building on this, the present study aims to investigate the underlying transcriptomic mechanisms (Bonnin et al., 2023). Breeding programs were designed for decades to produce new salt-tolerant rootstocks. Indeed, to cope with biotic and abiotic constraints, citrus trees are grafted onto rootstocks selected for their adaptability (Nawaz et al., 2016; Caruso et al., 2020).

Rootstocks are distinguished from scion varieties of agronomic interest by their intended use (Barry et al., 2020). Scion varieties are selected for the organoleptic qualities of their fruit, fruit size and yield. Rootstocks are chosen above all for their resistance to disease, pests, climate and soil type. Grafting a variety of agronomic interest onto a specific rootstock improves the graft’s chances of survival (Caruso et al., 2020). The increase in salinity associated with climate change calls for new rootstocks that are better adapted to salt stress. Sour orange (C. *aurantium* L.) was identified as an excellent Na^+^ excluder with good Cl^-^ exclusion capacity (Zekri, 1991). It was the main citrus rootstock worldwide during the first half of the 20^th^ century. Unfortunately, because of the susceptibility of this genotype to citrus Tristeza virus (CTV), it was necessary to move to other rootstocks with CTV resistance. Trifoliate orange (*P. trifoliata* [L.] Raf.) (PO) and its hybrids (citranges and Swingle citrumelo) are CTV tolerant rootstocks (Barry et al., 2020; Bowman and Joubert, 2020). However, they are unfortunately classified as sensitive to salinity (Khoshbakht et al., 2014). When grafted, PO is characterized by a superficial root system, making it sensitive to drought (Khoshbakht et al., 2014). It has also been described as sensitive to alkalinity (Khoshbakht et al., 2014). Poncirus hybrids such as Carrizo citrange (*Poncirus trifoliata x Citrus sinensis*) and Swingle 4475 citrumelo (*Poncirus trifoliata x Citrus paradisi*) are also commonly used as rootstocks. They share with their Poncirus parent tolerance to CTV and sensitivity to chlorides and drought. Many studies suggested that stress tolerance might be heritable traits (Storey and Walker, 1998; Singh et al., 2003; Flowers, 2004; Hussain et al., 2012; McGowan et al., 2021). Among *Citrus* species, Cleopatra mandarin (*C. reshni* Hort. Ex Tan.) (CL) has been identified as an excellent parental genotype in breeding programs because it has excellent salt stress tolerance properties (Zekri, 1991; Moya et al., 2003; Hussain et al., 2012; Raga et al., 2016). Used as rootstock, it is also tolerant to cold, drought, chlorides and limestone.

Increasing evidence suggests that polyploid citrus could have better environmental stress tolerance (Grosser et al., 2015; Oustric et al., 2019; Wei et al., 2020; Sivager et al., 2021; Lourkisti et al., 2022; Bonnin et al., 2023). An individual with more than 2n copies of chromosomes is polyploid. With a few exceptions, such as the triploid Persian lime (Ahmed et al., 2020), citrus are generally diploid (2x), having nine homologous chromosomes (n = 9) (Krug, 1943).

Previous work we performed (Bonnin et al., 2023) showed the interaction between genotypes and the ploidy level, allowing behaviour discrimination in terms of photosynthetic and antioxidant capacities under salt stress. Ploidy level enhanced PO’s and CL’s capacity to maintain photosynthetic activity under salt stress and enhanced their antioxidant capacities. Therefore, we concluded that tetraploidy may enhance salt stress tolerance in citrus. The objective of this study is to elucidate the transcriptomic mechanisms underlying the enhanced salt stress tolerance observed in tetraploid citrus genotypes compared to their diploid counterparts (Bonnin et al., 2023).

Polyploidization occurs during mitosis or gamete-formation mechanisms’ malfunction. Studies on citrus at the genomic, transcriptomic (Snoussi et al., 2012; Wei et al., 2020) and epigenetic levels have focused on the general pattern of neoregulation of the transcriptome (mRNA) (Xie, 2018; Song et al., 2023). So far, genes identified as important in salt tolerance are diverse and fall into the categories of osmoprotectant production, transcription factors involved in the regulation of growth and ion transporters and compatible solutes (Bartels and Sunkar, 2005; Ahmad et al., 2013; AbdElgawad et al., 2016; Acosta-Motos et al., 2017). This study aims to address the gap in knowledge of current genes involved in citrus salt stress tolerance, revealing genes involved in cell wall modification and antioxidant responses.

## 2 Materials and methods

### 2.1 Plant material and salt stress experiment

A transcriptomic experiment was performed on the same plant material and experimental design described in Bonnin et al. (2023). Briefly, four free-standing citrus genotypes were selected (**Supplementary Table S1**). Diploid (2x) trifoliate orange (*P. trifoliata*) (Poncirus Pomeroy ICVN-0110081) (PO2x) and 2x Cleopatra mandarin (*C. reshni*) (ICVN-0110274) (CL2x) and their two doubled diploid counterparts, 4x trifoliate orange (*P. trifoliata*) (ICVN-01011106) (PO4x) and 4x Cleopatra mandarin (ICVN-0101110) (CL4x) were evaluated in leaves and roots under salt stress conditions. Even if our study aim to select rootstock better adapted to salt stress, leaves data can provide a more comprehensive overview of the genetic regulation mechanism of salt stress. Briefly, plants selected for experimentation were divided into two blocks and watered once a week. Stressed plants were watered with a nutrient solution supplemented with salt (NaCl), and control plants were irrigated with nutrient solution only. Salt concentrations were steadily increased from 30 mM to 90 mM at increments of 20 mM per week and then stabilized at 90 mM.

### 2.2 Sample preparation and sequencing

Leaves and roots of the four citrus seedlings genotypes under the control and stressed conditions (90mM NaCl) were ground to a fine powder in liquid nitrogen under the RNAse-free condition. RNA extraction was performed using the NucleoMag® kit (Macherey-Nagel GmbH & Co.KU, Düren, Germany) with the KingFisher automated system (KingFisher Flex Purification System, ThermoFisher Scientific, Waltham, MA) according to the manufacturer’s protocol. RNA integrity and quality were checked on TapeStation, screentape D5000. RNAseq libraries were prepared to obtain labelled and matched sequences using UDI indexes, following the protocol and recommendations from the Illumina Truseq kit. The integrity and quality of the libraries were checked on TapeStation, screentape D5000 and assayed by qPCR. A first sequencing of the libraries was then performed to validate their quality using the Illumina MiSeq sequencing system at the Agap genotyping platform (Cirad, Montpellier). The equimolar pool of the libraries was then built up using the TruSeq Stranded mRNA Sample Preparation kit from Illumina. Sequencing was performed on a 150-nt pair-end S4 flow cell lane by MGX in Montpellier. The clustering and sequencing steps were performed on an Illumina NovaSeq 6000 using the SBS (Sequence By Synthesis) technique with NovaSeq Reagent Kits using the Illumina TruSeq RNA protocol (Illumina Inc., San Diego, CA, USA).

### 2.3 Data quality control and analysis

A total of 2.135.095.822 pairs of paired end reads of 150 nucleotides were generated from an Illumina Novaseq 6000 for the 48 samples with a mean of 44.481.163 read-pairs per samples. The rate of trimmed base was 8.79% and after low length reads removal, 2.122.477.755 (99.4%) of the initial read pairs remain available for mapping. 95.4% of these reads were successfully mapped on the reference genomes using STAR, with a multimapping rate of 3.48%. 86.3% of the mapped reads were asigned to knows features and were used for counting. More details can be found in **Supplementary Table S2** and **Supplementary Figures S5**, **S6**.

The quality of raw data from high-throughput sequencing was assessed by calculating different metrics, such as the quality of the sequences per base using the FastQC software (v.0.11.9). Our experimental design is described as follows: for each tissue (roots or shoots)/2 independent genotypes/2 ploidy levels/2 modalities (control/stress)/3 biological replicates (**Supplementary Table S1**). RNASeq sequences of 2x and 4x Poncirus trifoliata (PO2x and PO4x) were aligned on the PO as reference genome (Peng et al., 2020), while 2x and 4x Cleopatra mandarin (CL2x and CL4x) were aligned with CL genome (Droc et al., in press^1^). Cutadapt [v3.5; (Martin, 2011)] was used to find and remove Illumina Trueseq adapter sequences, remove low-quality base pairs in 5’ and 3’ of each read based on a quality cut-off of 30 and discard resulting pairs of reads containing a read shorter than 50 bases. Poly-g tails were also removed using the nextseq-trim option with a cut-off of 20. STAR (2.7.3a) (Dobin et al., 2013) was used with default parameters to align the reads on the reference genomes. To obtain count matrices, featureCount function of the Subread tool was used (release 2.0.1, 10 May 2023), with the options -M and –fraction to consider multi-mapped reads (Liao et al., 2013; Stark et al., 2019; Amarasinghe et al., 2020).

### 2.4 Statistical analysis for gene selection

Gene counts from each genotype and tissue were analysed using DESeq2 R package (Love et al., 2014). Genes with expression-level changes depending on the treatment, the ploidy level, or the interactions between the two factors, were selected using the likelihood ratio test (LRT), following the multifactorial design protocol in the DESeq2 R package as described in Favreau et al. (2023). Briefly, the LRT was applied to simultaneously test all the treatments and levels according to the multifactorial model Stress + Ploidy + Stress x Ploidy. Significant genes were selected at false discovery rate (FDR)-corrected *p*-values < 0.01 threshold. To evaluate the quality of the LRT selected genes and to explore the underlying structure of the selected gene set, partial least square discriminant analysis (PLSDA) was run using the Mixomics R package (release 3.17) (Lê Cao et al., 2011; Rohart et al., 2017). For each gene set, from the two genotypes and two tissues, three lists of genes were extracted: those responding only to each specific effect, ploidy and stress, and those responding to the interaction ploidy x stress.

Co-expression analysis was performed on the significant genes, selected according to the multifactorial model. The coseq R package (Rau and Maugis-Rabusseau, 2018) was used through the DIANE (Cassan et al., 2021) package with a normal model, an arcsin data transformation, a number of clusters between 3 and 20 and a seed fixed to 100.

The non-parametric Kruskall Wallis test was used to determine whether, for a given set of groups, there was at least one that was different from the others When KW test was able to identify d differences between groups, it was required to know which variables was concerned. We therefore carried out a pairwise multiple comparison, by using Dunn’s test (Dunn, 1973). KW and Dunn use the same ranks assigned to individuals to carry out their treatments.

### 2.5 Functional categorisation

Functional analysis of the genes was performed. Gene ontology (GO) associated with each gene was found using a combination of Interproscan (Jones et al., 2014) and blast best hit (Ward and Moreno-Hagelsieb, 2014) against the Viridiplantae subset of Uniprot SwissProt and TremBL. GO enrichment was then performed using the Cytoscape plugin BiNGO (Maere et al., 2005). For each list of genes, significantly enriched GO terms were identified using a hypergeometric test and a multiple test correction using the Benjamini and Hochberg FDR correction with a significance level of 0.05 (Benjamini and Hochberg, 1995; Thissen et al., 2002; Reiner et al., 2003; Ferreira, 2007; Green and Diggle, 2007; Stephanie, 2015). Enriched GO terms having similar functions were clustered using the Enrichment Map and AutoAnnotate plugins of the Cytoscape software (Isserlin et al., 2014; Kucera et al., 2016). Default parameters were applied for each plugin.

## 3 Results

In this section, we pinpoint significant groups of genes with contrasting expression profiles under salt stress according to the ploidy level of each genotype, regulating specific biological functions. More specifically, our findings highlighted six categories of genes that are enriched in photosynthesis, sugar metabolism, cell wall remodelling, ROS detoxification, gene expression regulation and signalling.

### 3.1 Data structure analysis

#### 3.1.1 Discriminant analysis

**Figure 1:**
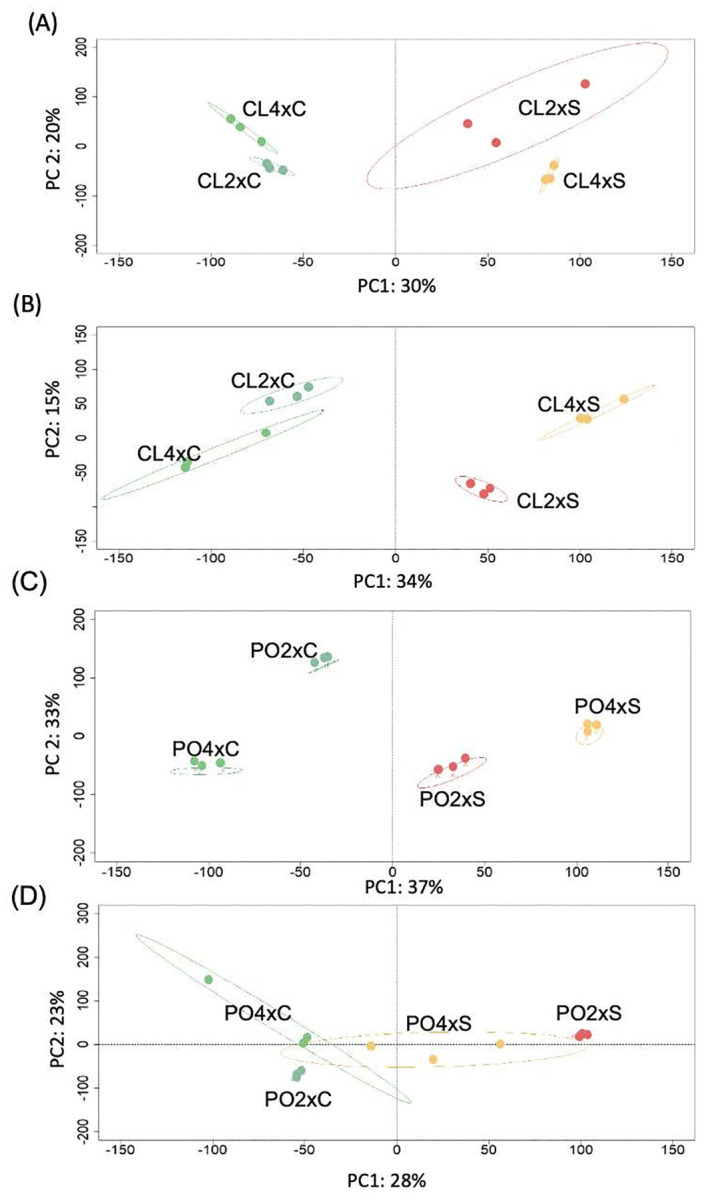
PLS-DA sample plot of transcriptomic data from of Cleopatra mandarin (CL) and Poncirus trifoliata (PO) genotypes with two ploidy levels (2x, 4x) in CL leaves **(A)**, CL roots **(B)**, PO leaves **(C)** and PO roots **(D)**. Samples are coloured according to genotype and conditions (green = control 2x and 4x, red=stressed 2x, orange = stressed 4x). Confidence ellipses were set to 95%. Measurements were performed after four weeks of salt stress (90 mM NaCl). C and S represent Control and Stressed plants, respectively.

Quality and structuration of CL and PO datasets were assessed thanks to independent PLS-DA analysis (**Figure 1**). For both CL tissues, salt treatment was the first factor that discriminated the gene expression profiles between the samples. The first principal component (PC1) explained 30% and 34% of the variability in leaves (**Figure 1A**) and roots (**Figure 1B**), respectively. Ploidy level was discriminated by PC2, representing 20% and 15% of the variability in leaves (**Figure 1A**) and roots (**Figure 1B**), respectively. In CL leaves, PC3 (14% of the variability) discriminated CL2x control (CL2xC) from other genotypes (**Supplementary Figure S1A**). In roots, PC3 (12% of the variability) discriminated the stressed CL2x (CL2xS) from the other genotypes (**Supplementary Figure S1B**). For PO, salt stress was the first factor that discriminated the leaf samples (PC1 = 37%; **Figure 1C**), and PC2 discriminated the two ploidy levels (33% of the variability). In PO leaves, however, PC3 (8% of the variability) discriminated the stressed PO2x (PO2xS) (**Supplementary Figure S1C**) from the other genotypes. In PO roots (**Figure 1D**), control and stressed treatments were discriminated on PC1 (28% of the variability), and ploidy levels were discriminated on PC2 (23% of the variability) for the control only. Moreover, in PO roots, PC3 (10% of the variability) discriminated between control PO2x (PO2xC) and the stressed PO4x (PO4xS) on one side and the other genotypes (PO4xC and PO2xS) on the other side.

In both tissues, for both PO and CL genotypes, salt stress is the primary factor discriminating gene expression profiles between samples. Ploidy level is discriminated on PC2, except in PO roots where ploidy level is discriminated only for controls. These results demonstrate that the interaction between ploidy level and salt treatment influences data variability. Based on this result, three independent gene lists were created. The absence of a common gene between the gene lists suggests that in 4x, different signalling pathways and biological targets could be regulated by stress, ploidy and/or the effect of the interaction between stress and ploidy.

#### 3.1.2 Identification of genes regulated according to the ploidy level, the salt stress or the interaction between ploidy and the salt stress

Gene expression was investigated for each genotype and each tissue. More specifically, the factors influencing the variation of the gene expression were identified (*i.e*., the ploidy level, the stress application or the interaction between both). Among the regulated genes in CL leaves (**Supplementary Figure S2A**), 4% of the genes were regulated according to the ploidy level only, 79% according to the treatment applied and 18% according to the interaction between ploidy and stress application (**Table 1**). In CL roots (**Supplementary Figure S2B**), 9%, 82% and 9% the genes were regulated according to the ploidy, the salt stress and the interaction between ploidy and stress, respectively (**Table 1**). In PO leaves (**Supplementary Figure S2C**), 26%, 34% and 40% the genes varied according to the ploidy, stress and the interaction between both, respectively (**Table 1**). In PO roots (**Supplementary Figure S2D**), only 15 genes were regulated according to ploidy level (1% of the total), while 81% and 19% varied according to the stress and the interaction between ploidy and stress, respectively (**Table 1**).

**Table 1:** Number of regulated genes influenced by the effect of stress, ploidy and the interaction of stress and ploidy.

Genotypes	Tissues	Stress	ploidy	Stress x ploidy
CL	Leaves	4049 (79%)	184 (4%)	905 (18%)
	Roots	5085 (82%)	530 (9%)	588 (9%)
PO	Leaves	2958 (34%)	2285 (26%)	3517 (40%)
	Roots	1615 (1%)	15 (81%)	317 (19%)

### 3.2 Identification of the biological processes regulated according to both stress and ploidy level

**Figure 2:**
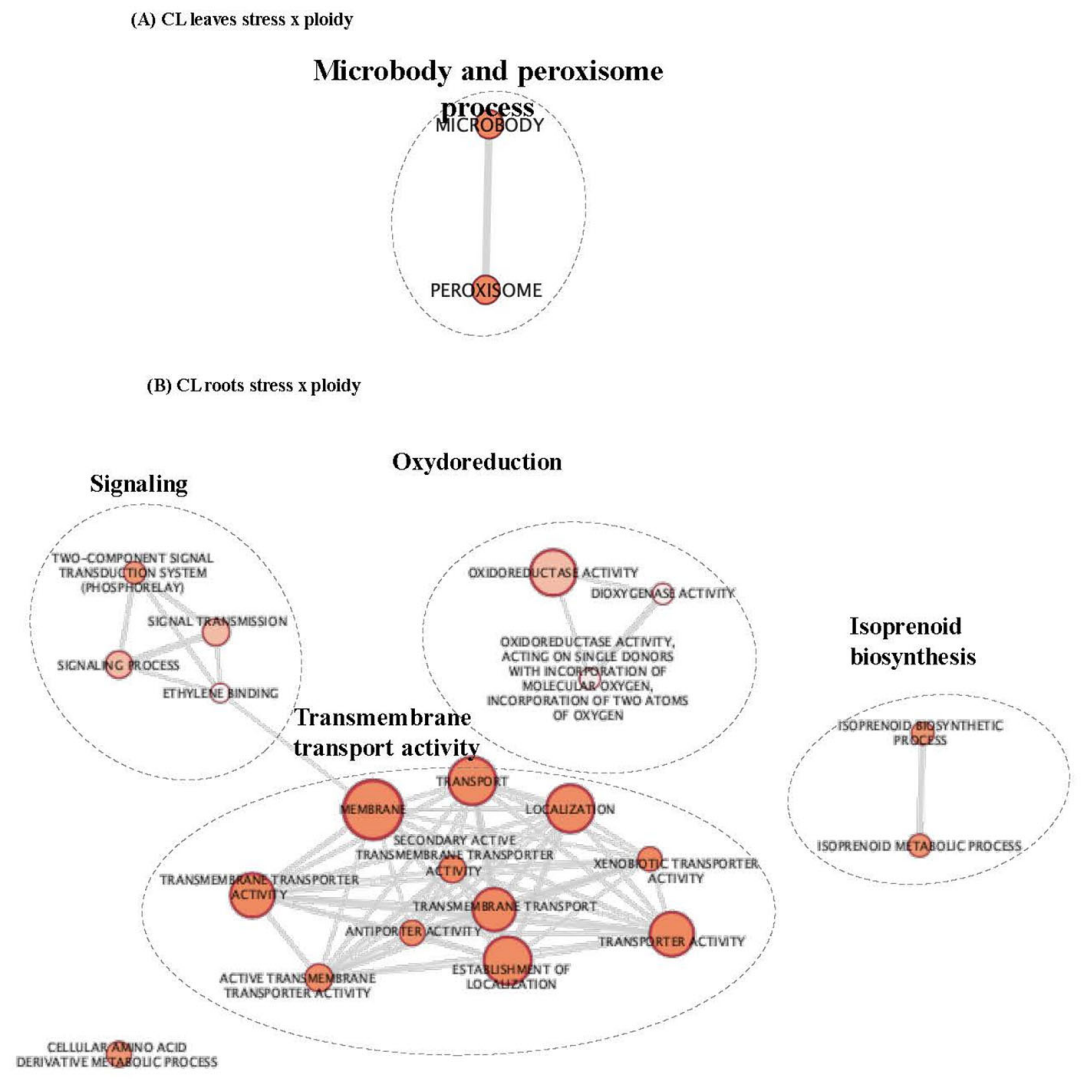
GO enrichment map of genes “stress x ploidy” DEGs in CL leaves **(A)** and roots **(B)**. Nodes represent enriched GO and edges between nodes indicate their functional similarity. Node size is proportional to the number of expressed genes. Intensity of node orange coloration corresponds to adjusted p-value (p-adjust). Edges length between nodes is proportional to the degree of functional similarity between them. Nodes having very similar biological functions were clustered and labelled with a summarized name.

**Figure 3:**
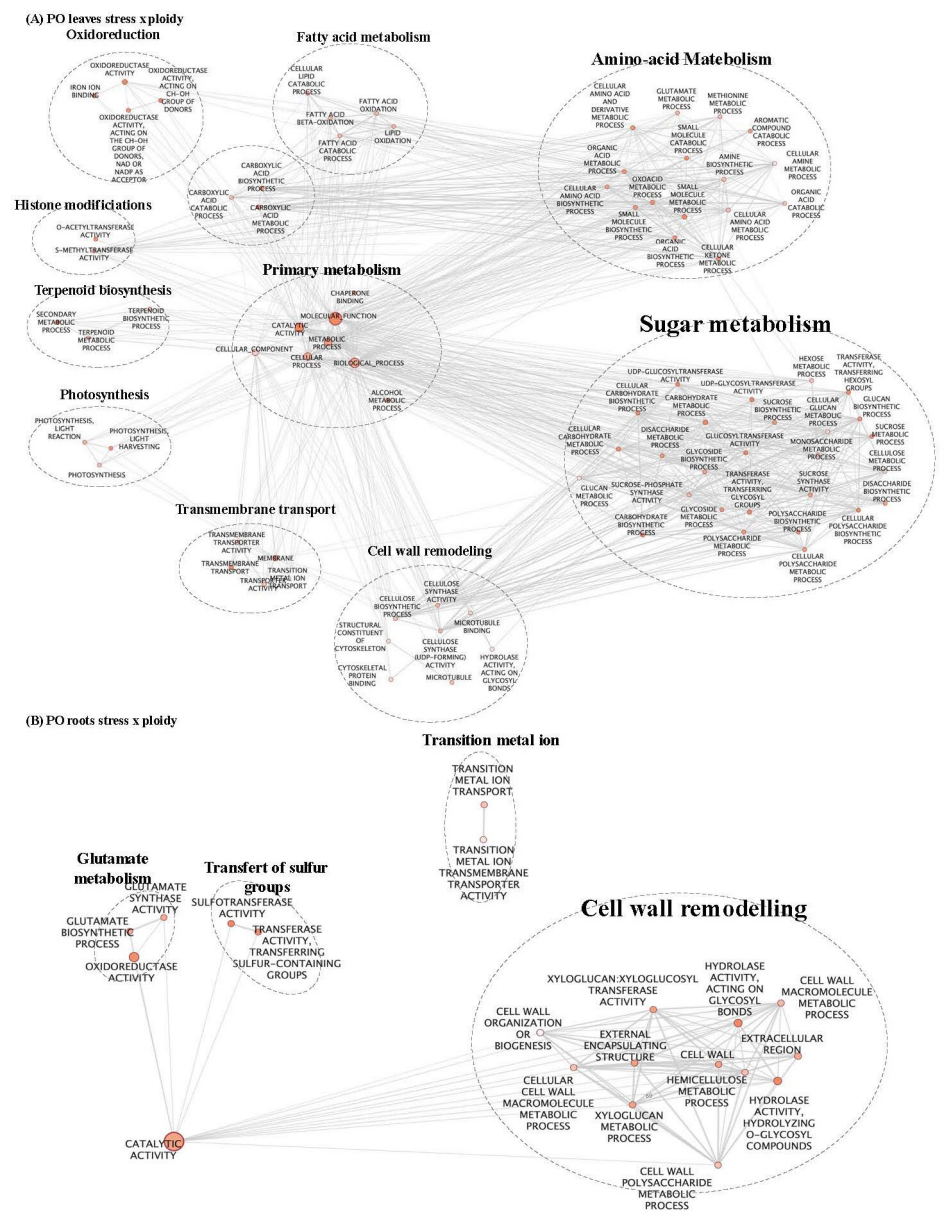
GO enrichment map of genes “stress x ploidy” DEGs in CL leaves **(A)** and roots **(B)**. Nodes represent enriched GO and edges between nodes indicate their functional similarity. Node size is proportional to the number of expressed genes. Intensity of node orange coloration corresponds to adjusted p-value (p-adjust). Edges length between nodes is proportional to the degree of functional similarity between them. Nodes having very similar biological functions were clustered and labelled with a summarized name.

**Figure 4:**
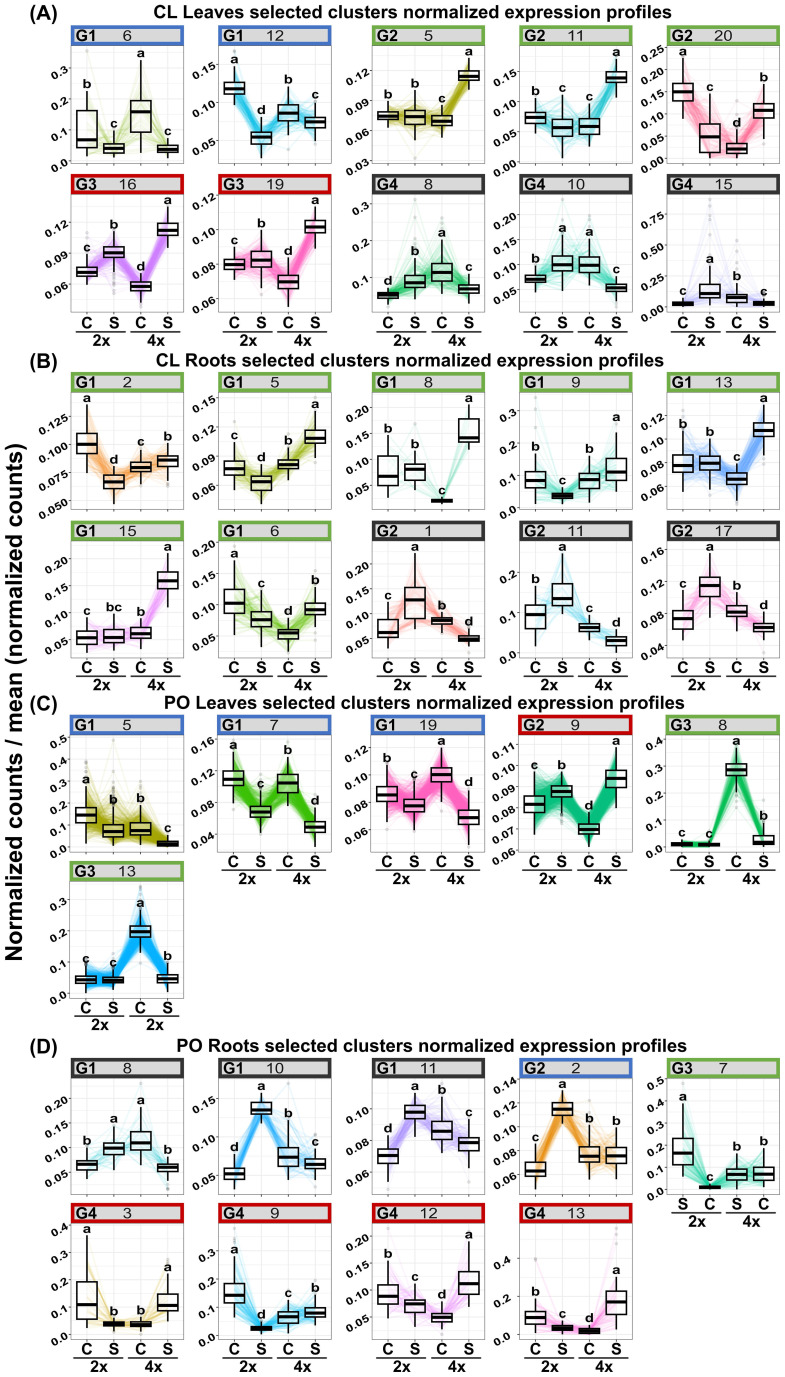
Clusters of co-expressed genes (p-value<0.01) “ploidy × stress” in 2x and 4x CL leaves **(A)** and roots **(B)** and PO leaves **(C)** and roots **(D)** under control (C) and salt treatment (S). Significance (p-value<0.01). Clusters with no enriched GO terms were identified by a grey square. Enriched clusters identified by a black star and sharing the same expression profile were classified in clusters groups. In CL Leaves **(A)**, groups 1 clusters (G1) are identified in blue. Group 2 clusters (G2) were identified in green. Gene clusters belonging to the group 3 (G3) are up-regulated in both 2x and 4x in stress compared to the control. Genes of these clusters are highlighted in red. Genes belonging to the group 4 (G4) are identified in black. CL roots **(B)** genes from group 1 (G1) are highlighted in green. Clusters belonging to group 2 (G2) are represented in black.

PLS-DA analysis highlighted that, for each genotype and tissue, stress treatment differentially modified gene expression depending on the ploidy level. Therefore, to identify the biological processes that differentiate the response of each ploidy level to salt stress, GO enrichment was performed on the gene list regulated by “ploidy x treatment” (**Table 2**; **Figures 2**, **3**; **Supplementary Table S2**). To deepen the previous analysis, we searched for groups of genes, and corresponding biological processes, that could characterise each ploidy level’s specific response to salt stress. Therefore, gene co-expression was analysed in the list “ploidy × treatment” for each genotype and tissue, and clusters of genes having similar expression profiles were identified (**Figure 4**).

**Table 2:** Summary of the main biological processes identified in the list of regulated genes “stress × ploidy”, “stress” and “ploidy”.

Genotypes	Tissues	Ploidy x Stress	Stress	Ploidy
CL	LEAVES	Microbody peroxysome	Primary metabolism Glycolytic process Photosynthesis Protein phosphorylation Aminoacid binding	ADP binding
	ROOTS	Signalling, Oxidoreduction Transmembrane transport activity Isoprenoid biosynthesis	Nucleotide biosynthesis Protein localization Protein biogenesis Regulation of primary metabolism	Signalling process Transcriptional Regulation of primary metabolism regulation of ROS response Organic acid transport Transmembrane transporter activity
PO	LEAVES	Primary metabolism Sugar Metabolism Amino acid metabolism Fatty acid metabolism Carboxylic acid metabolism Histone modification oxidoreduction Terpenoid biosynthesis Photosynthesis Transmembrane transport	Regulation of transcription and translation Negative regulation of organelle organisation	Post-translation regulation
	ROOTS	Cell wall remodelling, Glutamate metabolism Transition metal ion Transfer of sulfur groups	Ribonucleoprotein complex involved in gene expression Nucleotide binding regulation of circadian rhythm	Regulation of photosystem II by chlorophyll binding Nucleotide binding Structural constituent of cell wall

In CL leaves, peroxisome and microbody processes were differentially regulated between 2x and 4x under salt stress (**Figure 2A**; **Supplementary Table S2**), and in CL roots, signalling and transport functions differed between the two ploidy levels (**Figure 2B**; **Supplementary Table S2**). In (CL), 20 clusters in leaves (**Figure 4A**) and 18 in roots (**Figure 4B**) were identified. In the PO genotype, 20 and 14 clusters were identified in leaves and roots, respectively (**Figures 4C, D**). For each tissue and genotype, clusters were classified by trends: genes up- or down-regulated for 2x and 4x between control and stressed plants. In CL leaves, four groups of clusters are identified (**Figure 4A**). Genes belonging to group 1 (clusters n°6 and 12) are downregulated in both 2x and 4x under salt stress compared to the control treatment, but at a different amplitude for each ploidy. These genes are mostly involved in photosynthesis (**Supplementary Table S2**). Gene expression from group 2 (clusters n°5, 11 and 20) increases in stressed 4x, compared to the control, and shows no variation of expression or is down-regulated in stressed 2x, compared to the control. Corresponding genes are associated with trehalose biosynthesis, K^+^ transport and cell wall remodelling. Genes from group 3 (clusters n°16 and 19) are up-regulated in both 2x and 4x under stress compared to the control at a different amplitude according to the ploidy level. They are related with ion transport and peroxisome processes (**Supplementary Table S2**). Genes from group 4 (clusters n°8, 10 and 15) are up-regulated in 2x but do not vary or are down-regulated in 4x under salt stress, compared to the control. They are mostly associated with chitin and antioxidant metabolism, cell wall remodelling and microbody processes (**Supplementary Table S2**). Similarly, in CL roots, on 18 clusters selected previously, only ten presented GO enrichment (**Supplementary Table S2**). GO-enriched clusters could be classified into two groups of expression profiles: genes from group 1 (clusters n°2, 5, 6, 8, 9, 13 and 15) having no variation of expression or presenting down-regulation for 2x, while they are up-regulated in the 4x for stressed plants compared to the control (**Figure 4B**). They are mostly associated with sterol and steroid biosynthesis, transmembrane transport (K^+^ transport, passive and active transport) and signalling (**Supplementary Table S2**). Clusters belonging to group 2 (clusters n° 1, 11 and 17) contain genes that are up-regulated in stressed 2x and down-regulated in stressed 4x compared to the control (**Figure 4B**). They are related to cellulose and polysaccharide biosynthesis, chitin metabolism, post-transcriptional regulation (RNA-processing) and signalling (**Supplementary Table S2**).

In both CL and PO, there are groups of under-expressed genes, whatever the ploidy level considered under salt stress conditions. The genes in these groups share GO terms related to the photosynthesis process. This result is in line with the findings of the previous chapter, showing a disruption of photosynthesis processes in all the genotypes tested.

In PO leaves, among the number of enriched GO terms, we identified GO terms linked to sugar biosynthesis, photosynthesis and cell wall remodelling (**Figure 3A**; **Supplementary Table S2**). Cell wall organisation and biogenesis were regulated in PO roots (**Figure 3B**; **Supplementary Table S2**). Altogether, these results reveal that under salt stress, cell wall remodelling, sugar biosynthesis, photosynthesis, signalling and regulation of gene expression were differentially regulated between the two ploidy levels. In PO leaves, nine clusters had enriched GO (**Supplementary Table S2**) that could be classified into five groups of expression patterns. Group 1 (clusters n°5, 7 and 19) presents genes down-regulated in stressed plants, compared to the control, with different amplitudes in 2x and 4x. They are enriched in GO terms associated with ROS metabolism, cell wall remodelling, photosynthesis and ion transport (**Supplementary Table S2**). Group 2 (clusters n°9) contains genes up-regulated in 2x and in 4x stressed genotypes at different amplitudes compared to the control (**Figure 4C**). They are mostly associated with post-transcriptional regulation (RNA-splicing). Group 3 (clusters n°8 and 13) is characterised by a down-regulation in CL4xS compared to its control, while gene expression does not vary in 2x (**Figure 4C**). They are mostly associated with cell wall remodelling, enzyme activity regulation (**Supplementary Table S2**; **Figure 4C**), post-transcriptional regulation (RNA-processing) and epigenetic regulation (histone modification) (**Supplementary Table S2**). Finally, cluster analysis on PO roots highlighted eight GO-enriched clusters (**Supplementary Table S2**) that could be classified into four groups of expression patterns.

Group 1 (clusters n°8, 10 and n°11) is composed of down-regulated genes in PO4xS compared to PO4xC; these genes are up-regulated in PO2xS compared to PO2xC (**Figure 4D**). They are principally related to photosynthesis (**Supplementary Table S2**). We found another group of genes in PO leaves that were underexpressed at all ploidy levels. This time, these genes were linked to carbohydrate metabolism. In CL, genes whose functions are linked to carbohydrate metabolism do not have the same expression profile. Their expression does not vary in 2x, but increases in 4x under salt stress. Higher sugar levels in CL4x could therefore contribute to its better adaptation to salt stress.

In group 2 (cluster n°2), genes are up-regulated in 2xS compared to 2xC, with no variation observed in 4x (**Figure 4D**). They are mostly related to translation regulation (**Supplementary Table S2**). Genes belonging to group 3 (cluster n°7) are down-regulated in 2xS compared to the control, while they do not vary in 4x (**Figure 4D**). They are principally related to GO terms linked to cell wall remodelling and transcription factor activity (**Supplementary Table S2**). Group 4 (clusters n°3, 9, 12 and 13) genes are down-regulated in 2xS compared to 2xC. Conversely, genes of these clusters are up-regulated in the corresponding 4xS compared to 4xC (**Figure 4D**). They are mostly related to hormone signalling (abscisic acid), antioxidant activity, ion transport and enzyme activity regulation (**Supplementary Table S2**). Among the clusters possessing genes overexpressed in 4x under salt stress, in contrast to their 2x counterparts, we also found genes linked to cell wall remodelling and transport activities and maintenance of ionic homeostasis. Overall, our results show that in both CL and PO, the level of ploidy influences transport management under salt stress. Enhanced ion storage or exclusion could improve salt stress tolerance in 4x genotypes.

### 3.3 Identification of genes regulated according to ploidy level or stress

**Figure 5:**
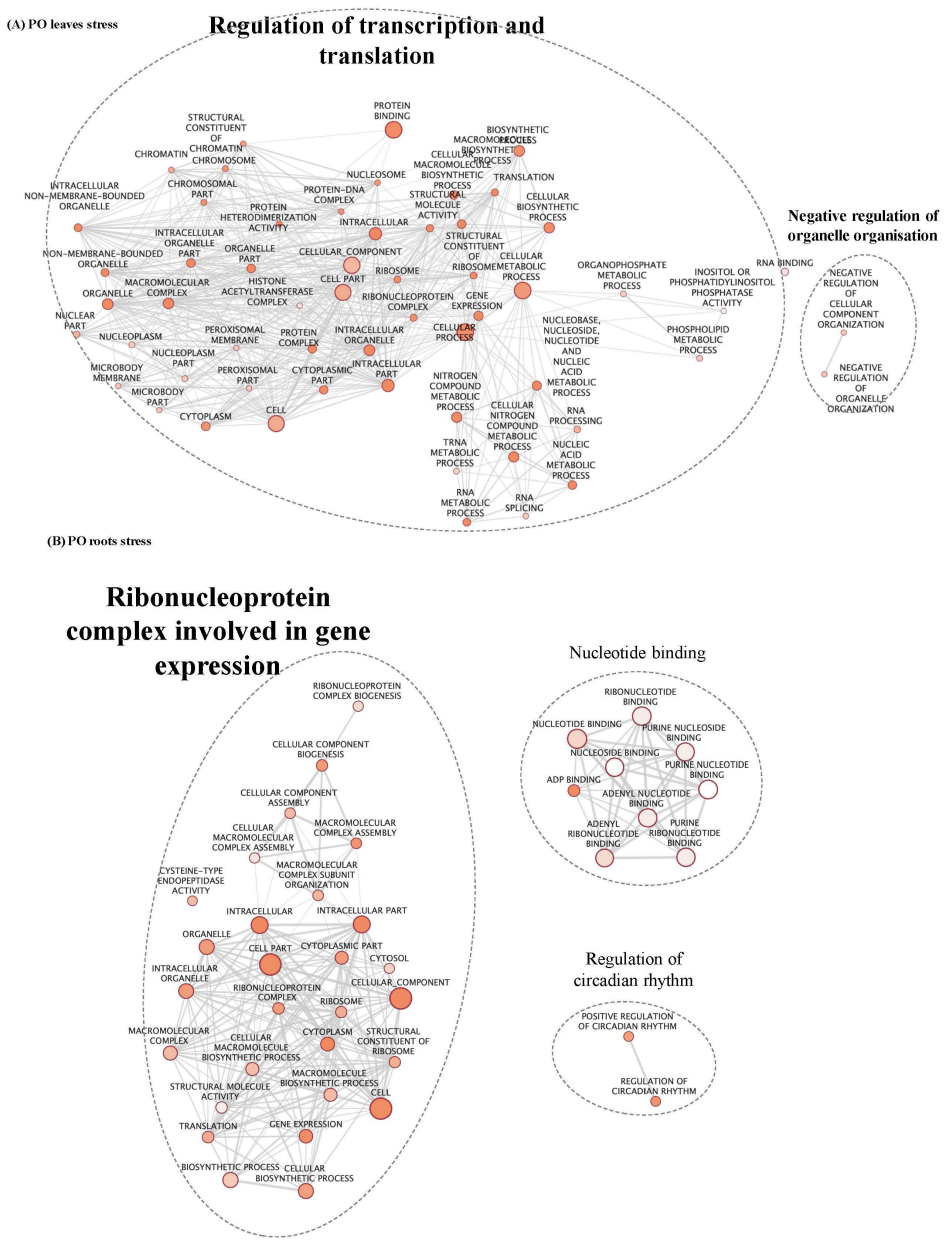
GO enrichment map of genes “stress” in PO leaves **(A)** and roots **(B)**. Nodes represent enriched GO and edges between nodes indicate their functional similarity. Node size is proportional to the number of expressed genes. Intensity of node orange coloration corresponds to adjusted p-value (p-adjust). Edges length between nodes is proportional to the degree of functional similarity between them. Nodes having very similar biological functions are clustered and labelled with a summarized name.

Beside genes influenced by ploidy and stress, it was interesting to identify the molecular mechanisms that were influenced by each factor regardless of the other. First, GO enrichment was performed on both lists of genes, “ploidy” and “stress”, and a map enrichment of GO terms designed. GO terms were summarised in clusters including GO belonging to similar pathways and processes, representing main biological mechanisms (**Table 2**). In CL leaves, GO terms identified in genes regulated under stress treatment are principally related to glycolytic process, primary metabolism, photosynthesis, amino-acid binding and protein dephosphorylation (**Table 2**; **Supplementary Figure S3A**). In CL roots, genes are mostly associated with nucleotide biosynthesis, protein localisation, protein biosynthesis and regulation of primary metabolism (**Table 2**; **Supplementary Figure S3B**; **Supplementary Table S2**). In PO leaves, an important number of genes linked to regulation of transcription and translation (**Table 2**, **Figure 5A**) were identified. In PO roots, genes involved in ribonucleoprotein (RNP) complex concerned with gene expression (**Table 2**; **Figure 5B**) as well as nucleotide binding and regulation of circadian rhythm were also found. Altogether, our results suggest that, in both PO and CL, salt stress treatment induced neoregulation for a large number of genes involved in reprograming, targeting photosynthesis, signalling and cellular biosynthetic process.

**Figure 6:**
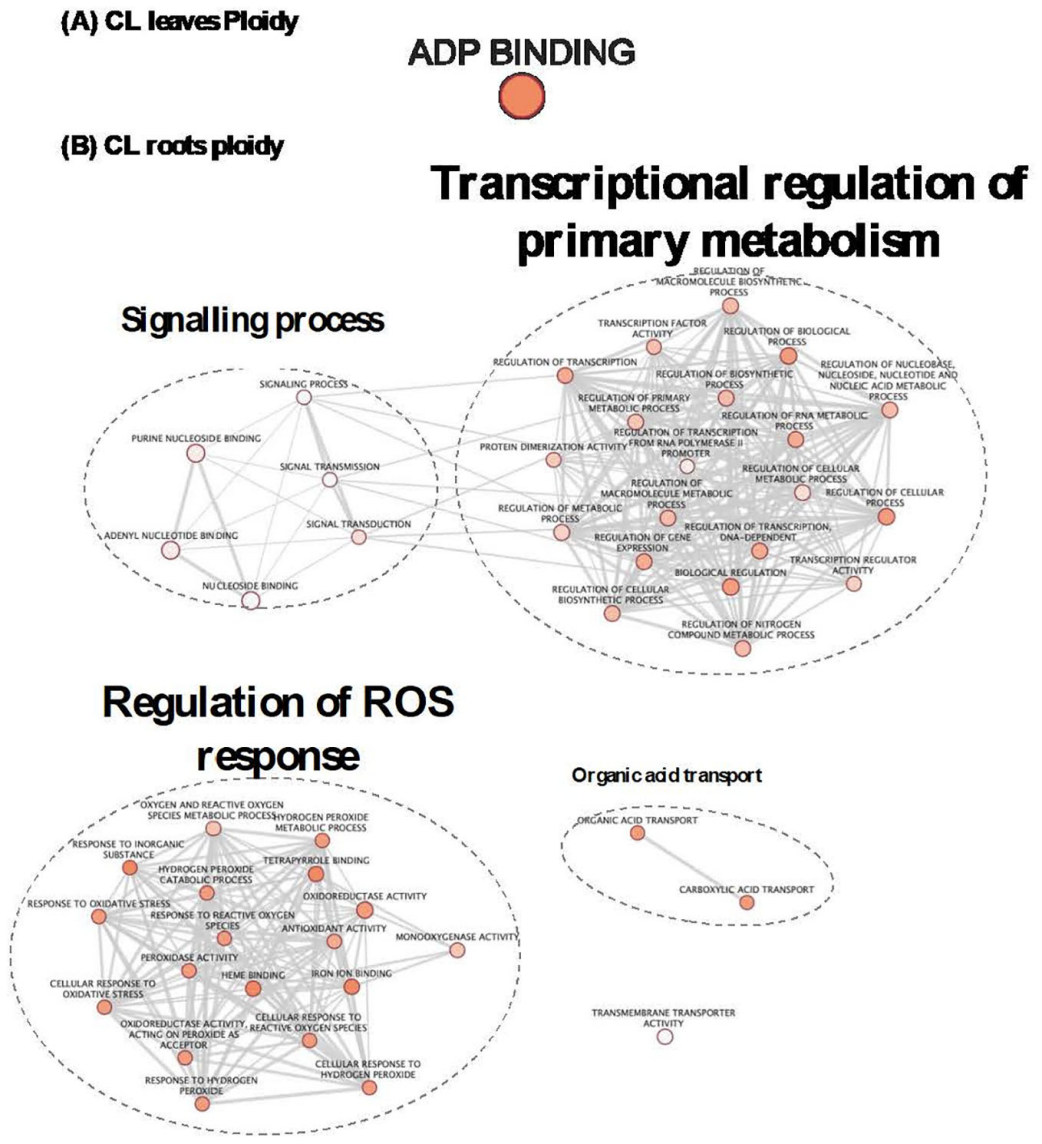
GO enrichment map based of ploidy DEGs in CL leaves **(A)** and roots **(B)**. GO enriched gene sets connected by edges mean that they shared a similarity. Nodes belonging to very similar gene function are clustered and labelled with a summarized name. Node size corresponds to enrichment significance (p-value). Intensity of orange coloration corresponds to adjusted (p-ajust). Node size is proportional to the number of expressed genes. Edges thickness between nodes is proportional to the degree of similarity between them.

To study the influence of the ploidy level on gene expression, irrespective of the treatment applied, GO enrichment analysis was performed with the list “ploidy” for each genotype (**Supplementary Table S2**). In CL leaves (**Table 2**; **Figure 6A**), the only enriched biological process that explained the difference between ploidy levels was the ADP binding process (**Table 2**; **Figure 6A**). In CL roots, the expression of a higher number of genes was influenced by ploidy level (**Table 2**; **Figure 6B**; **Supplementary Table S2**). DEGs related to ploidy were mostly involved in signalling, transcriptional regulation of primary metabolism, regulation of ROS response, organic acid transport and transmembrane transport activity (**Table 2**; **Figure 6B**). This suggests a possible role of ploidy level in signalling, gene expression reprograming and antioxidant defence systems in CL roots.

Strikingly, in PO leaves, genes regulated according to ploidy level (**Supplementary Figure S4A**) are mostly related to post-translational regulation. Finally, in PO roots (**Supplementary Figure S4B**), three clusters of gene sets were identified. The most important ones are related to regulation of photosystem II by chlorophyll binding, nucleoside metabolism and structural constituents of cell walls.

### 3.4 Identification of genes regulated according to ploidy level under control conditions

In CL roots, genes regulated according to ploidy level under control conditions (**Supplementary Table S2**) are mostly related to post-translational regulation and response to oxidative stress. No GO enrichment was detected in CL leaves under control conditions when focusing on genes strictly influenced by ploidy level. In PO roots under control conditions, genes strictly influenced by ploidy level are related to ADP binding. Similarly, in PO roots, we identified genes mostly related to sugar and nucleotide metabolism (**Supplementary Table S2**).

## 4 Discussion

In several studies, chromosome set doubling was shown to provide better adaptation to different abiotic stresses, including salt stress (Ruiz et al., 2020; Bonnin et al., 2023). Relevant crops include citrus (Ruiz et al., 2020), watermelon (Zhu et al., 2018), and soybean (Mangena, 2023). These studies showed that a whole-genome duplication has a strong, genome-wide effect on gene expression and that the transcriptional response to stress varies between 4x and 2x. Our previous work demonstrated that in PO and CL, 4x displayed enhanced salt tolerance compared with 2x (Bonnin et al., 2023). Here, we emphasised the gene expression determinant of salt stress tolerance in these two genotypes, comparing 4x *vs* 2x plants. We characterized the response to salt stress of our genotypes into six gene-function classes: (1) photosynthesis, (2) osmoprotection, (3) cell wall remodelling, (4) transporters, (5) ROS detoxication and (6) expression regulators and signalling pathway-related genes. We choose to include leaves data in this a transcriptomic evaluation focused on rootstocks selection. In this context, the genetic regulation of leaves is less significant compared to the root system. Still leaves data can provide a more comprehensive overview of the genetic regulation mechanism of salt stress. In a previous study we evaluated the decrease in leaf chlorophyll content in salt-stressed citrus by monitoring SPAD measurements throughout the stress period (Bonnin et al., 2023). The decrease in SPAD values is only significant for sensitive PO2x. Numerous studies have also reported that salt stress decreases chlorophyll content in sensitive genotypes (Khoshbakht et al., 2014; Hameed et al., 2021). However, no leaf symptoms were observed in the leaves of CL, nor any variation in SPAD value under salt stress. Similar to what we observed, Ben Yahmed et al. (2015) demonstrated that mandarin accessions such as ‘Cleopatra’ and ‘Shekwasha’ showed few leaf symptoms. According to these authors, for these genotypes, Cl^-^ translocation from roots to leaves is limited. Accessions with the most severe foliar symptoms, such as ‘Fuzhu’, ‘Willowleaf’, ‘Beauty’, ‘King of Siam’ and ‘Nasnaran’, have higher Cl^-^ translocation from roots to leaves (Cl- leaf/root ratio). Other research (Brumós et al., 2009) has shown that, in CL mandarin, salt stress does not lead to the accumulation of toxic levels of chlorides in shoots. Finally, a study on the high-salinity-tolerant rootstock (Foral-5) (López-Climent et al., 2008) showed that the combination of an efficient Cl^-^ exclusion mechanism and an active photosynthetic system can improve salt stress tolerance in this genotype. An interesting perspective to our work would be to study the potential of CL and CR rootstocks grafted with varieties of commercial interest (such as orange or mandarin). Improved ion restriction capabilities at root level should alleviate symptoms caused by salt stress.

### 4.1 Better cell wall strengthening under salt stress in CL4x

In salt stress condition, as ROS concentration increases (Apel and Hirt, 2004; Ahmad et al., 2013; AbdElgawad et al., 2016; Bonnin et al., 2023), it can also initiate the formation of radicals that may cleave sugar bonds in plant polysaccharides, causing cell wall loosening. Cell wall integrity (CWI) maintenance is a factor of paramount importance for plants to tolerate perturbations in turgor and to reinforce cell walls in situations of high salinity (Feng et al., 2018; Colin et al., 2023). Reduced ability of plant cells to absorb water decreases cellular turgor pressure, thereby causing a retraction of plant tissues and extrusion of water from the plant cells (van Zelm et al., 2020). To overcome the osmotic stress, cell wall adjustment is required to adapt to reduced turgor pressure and altered CWI (Colin et al., 2023). Peroxidases can crosslink wall glycoproteins, such as extensins, as well as components of the secondary cell wall, such as lignins. The establishment of crosslinks between these components suggests strengthening of the cell wall. Activation of peroxidases involved in cell wall remodelling (Shen et al., 2014; Le Gall et al., 2015; Tenhaken, 2015; Novaković et al., 2018) could be a strategy to withstand osmotic changes caused by salt stress (Tenhaken, 2015). Many authors have noticed phenotypic differences between ploidy levels. These differences include increased cell volume and a more massive appearance of the organs (Ruiz et al., 2020). It has been demonstrated that leaves of polyploids could be thicker and exhibit greater photosynthetic activity and chlorophyll content than 2x (Ruiz et al., 2020; Sivager et al., 2021; Lourkisti et al., 2022; Tossi et al., 2022). Roots of 4x genotypes are generally thicker and less branched, with less root development than those of 2x (Tossi et al., 2022). It is believed that these histological characteristics of roots could be associated with lower hydraulic conductivity. Such an ability may be an advantage to cope with the effects of salt stress. This is why it is thought that 4x citrus genotypes, once used as rootstocks, could provide a sustainable system to cope with the toxicity problem caused by ion excess. Previous studies have already demonstrated the relationship between phenotypic variation in the aerial part and stress resistance in polyploid citrus (Moya et al., 2003; Allario et al., 2011; Ruiz et al., 2020). In contrast, the role of the root system is poorly documented (Bonnin et al., 2023). If the maintenance of turgid cells is mostly dependent on water influx, cell collapse due to plasmolysis is mostly related to the thickness of the cell wall and maintenance of its integrity (Colin et al., 2023). Plants defective in cell wall synthesis and remodelling are typically hypersensitive to salt stress. In our study, co-regulated gene clusters related to cell wall remodelling were identified in both PO and CL genotypes, potentially leading to cell wall modifications that might be relevant to salt response (Shen et al., 2014; Tenhaken, 2015; Byrt et al., 2018; Colin et al., 2023). In PO roots, gene clusters n°5 and 9 as well as gene cluster n°20 in CL leaves contained genes related to cell wall organisation, dynamics and modification (**Figure 4**; **Supplementary Table S2**). Interestingly, in these clusters, 4x genes were up-regulated under salt stress, contrary to their 2x counterparts. Genes identified in these clusters were encoded for expansin 1 (CITRE_004g032280; cluster n°20 CL leaves) and extensin 3 (Ptrif.0005s2249; cluster°9 PO roots). We also found genes encoding for pectin methylesterase 17 (Ptrif.0003s4304; cluster n°5 PO leaves) (**Supplementary Table S2**). Expansin proteins are known to have cell-wall loosening activity (Sampedro and Cosgrove, 2005). They could be involved in cell expansion and other developmental events during which cell-wall modification occurs (Sampedro and Cosgrove, 2005; Chen et al., 2018; Backiyarani et al., 2022). Extensins are plant cell wall hydroxyproline-rich glycoproteins (Castilleux et al., 2021). They are responsible for cell wall reinforcement by forming intra- and intermolecular cross-links (Zhao et al., 2018). It is thought that formation of such cross-links requires appropriate glycosylation and structural conformation of the glycoprotein (Showalter et al., 1992; Tiré et al., 1994; Lamport et al., 2011; Castilleux et al., 2021). Pectin methylesterases (PMEs) catalyse the removal of methyl groups from the homogalacturonan (HG) backbone (Hocq et al., 2017; Del Corpo et al., 2020; Gigli-Bisceglia et al., 2022). An abundant literature agrees that pectin is a major polysaccharide of cell walls rich in galacturonic acid (GalA) (Jarvis, 1984; Mohnen, 2008; Bethke et al., 2016; Hocq et al., 2017). Moreover, HG could be considered the most abundant pectic polymer in plant cell walls (Wydra and Beri, 2006; Yapo et al., 2007; Atmodjo et al., 2011; Hocq et al., 2017). HG could partially be methyl-esterified at the C6 atom of GalA (Micheli, 2001; Du et al., 2022). The degree and pattern of methylation could affect biomechanical properties of the cell wall by making pectin susceptible for enzymatic de-polymerization and enabling gel formation (Levesque-Tremblay et al., 2015). PMEs’ activity is modulated by a family of proteinaceous inhibitors known as pectin methylesterase inhibitors (PMEIs) (Levesque-Tremblay et al., 2015; Wormit and Usadel, 2018; Gigli-Bisceglia et al., 2022). In line with this, we found in cluster n°9 of PO roots a gene encoding for a PMEI (Ptrif.0005s0849; **Supplementary Table S2**). These results suggest that in CL leaves and PO roots, ploidy could improve cell wall maintenance and integrity during salt stress.

The CL4x strategy for cell wall remodelling could explain its improved salt tolerance. Moreover, recent work has demonstrated that ionic imbalance induced by salt stress could also impact cell walls’ mechanical properties (Favreau et al., 2019, 2023; Colin et al., 2023). Indeed, salt stress might cause cell wall softening, notably through its effect on pectins (Beauzamy et al., 2015; Feng et al., 2018; Favreau et al., 2019, 2023). Plant cell wall pectins are typically secreted in a highly methyl-esterified form and then selectively de-esterified by PMEs (Gigli-Bisceglia et al., 2022). When pectins are de-methylesterified, they can be cross-linked by divalent cations, such as Ca^2+^. This promotes cell wall stiffness (Hocq et al., 2017; Colin et al., 2023). Excessive Na^+^ concentration in the apoplastic region results in a competition with Ca^2+^ to bind to pectin, thereby disturbing the cross-linking of pectin (Ngouémazong et al., 2011).

### 4.2 Specific biological mechanisms triggered by salt stress

The lack of common genes between the gene lists (**Supplementary Figure S2**) suggests that ploidy, stress and interactions between both factors might trigger various responsive molecular mechanisms at the cell level (**Figures 6A, B**). This could mean that in 4x, different signalling pathways and biological targets might be regulated by stress, ploidy or stress–ploidy interaction effects. This also suggests that in 4x, specific genes’ expression is modified by functions of the synergetic effect of stress and ploidy. These genes can be discriminated from the specific stress- and ploidy-induced DEGs. In cluster n°9 PO leaves, genes were up-regulated under salt stress in 2x and 4x citrus at different amplitudes (**Figure 4C**). In this specific cluster, we noticed a certain number of GO terms related to epigenetic, transcriptional and post-transcriptional regulation (RNA-splicing). Alternative splicing (AS) is mediated by a large RNP complex known as the spliceosome. Spliceosomal pre-mRNA binding specificity is mediated by members of the serine/arginine-rich (SR) protein family. Many publications have already demonstrated that splice site selection could be involved in salt stress response (Ding et al., 2014; Filichkin et al., 2015; Feng et al., 2018). In line with this, we identified in PO leaves, cluster n°9, six genes related to the RNA-splicing function (Ptrif.0003s3334, Ptrif.0004s1700, Ptrif.0005s0128 and Ptrif.0005s0389, Ptrif.0009s2279, Ptrif.0002s2733), (**Supplementary Table S2**). These genes encode for PRP4 KINASE A (PRP4KA), MOS4-ASSOCIATED COMPLEX 7, MERISTEM-DEFECTIVE (MDF)/DEFECTIVELY ORGANIZED TRIBUTARIES 2 (DOT2), ATROPOS (ATO), SERRATE (SE) and SWELLMAP1 (SMP1) proteins, respectively. PRP4KA is a putative spliceosome protein kinase (Kanno et al., 2018). It is known to be an important for AS and plant development. MOS4 is a core member of the nuclear MOS4-associated complex (MAC) implicated in pre-mRNA splicing (Johnson et al., 2011; Jia et al., 2017; Tu et al., 2022). ATO is known to be the homolog of human SF3a60 and *S. cerevisiae* PRP9, which are required for the formation of the spliceosome complex (Moll et al., 2008; Staiger and Brown, 2013). The MDF DOT2 gene encodes an SR-related family protein required for meristem function and leaf vascularization. It is described as likely to be the homolog of the human SART1 and yeast snu66 splicing factor (Thompson et al., 2023). SE is a protein involved in the processing of RNA polymerase II (RNAPII) transcripts. It is associated with various complexes with roles in different aspects of plant RNA metabolism. These roles include assemblies involved in transcription, splicing, polyadenylation, miRNA biogenesis and RNA degradation (Raczynska et al., 2014; Jozwiak et al., 2023). mRNA processing SMP1 and SWELLMAP2 (SMP2) are involved in recruiting the second-step splicing factors to the spliceosome (Clay and Nelson, 2005; Liu et al., 2016). This suggests the possible activation of spliceosome complex in both types of PO under salt stress.

Interestingly, the PO leaves cluster n°9 also contained several genes associated with the epigenetic regulation process. Indeed, we found three genes related to histone modification of GO terms. These genes were the homolog of yeast ADA2 2A, SET domain protein 19, su(var)3-9 homolog 3 and plant homologous to parafibromin (Ptrif.0001s1025, Ptrif.0002s1614, Ptrif.0005s0808 respectively). ADA2 2A encodes for histone acetyl transferase GCN5 and the associate co-activator ADA2 (Vlachonasios et al., 2021). ADA 2A is part of the SAGA complex, which modifies nucleosomal histones through acetylation and other histone modifications (Moraga and Aquea, 2015). SET domain protein 19, su(var)3-9 homolog 3 is known to have methyltransferase activity. It generally targets specific lysine residues of histone H3 or H4 (Ng et al., 2007). Plant homologous to parafibromin is part of the PAF1 complex, which regulates gene expression within H3K27ME3-enriched chromatin (Park et al., 2010). These results could suggest that in polyploids, salt stress could trigger gene expression reprogramming through RNA splicing as well as epigenetic regulation.

### 4.3 Polyploidy enhanced specific biological mechanisms under salt stress

In PO leaves, genes strictly influenced by ploidy level were mostly related to post-translational regulation processes (**Supplementary Figure S4A**). In CL root genes strictly influenced by ploidy level, genes related mostly to signalling, transcriptional regulation and regulation of the ROS response (**Figure 6B**). This result suggests that ploidy may influence gene expression regulation. It can trigger various regulation mechanisms, and expressions of stress-responsive genes. In CL roots, our analysis was strengthened by the identification of specific genes. We identified various expansin-related genes (CITRE_006G018470, CITRE_006G018470 and CITRE_007G024720), an ascorbate-oxidase gene (CITRE_002G005950) and a putative CaM_binding domain-containing protein (CITRE_003G011190) (**Supplementary Data Sheet 1**). Expansins are known to be key regulators of cell-wall extension, involved in the abiotic stress response. Salt stress triggers the reduction of shoot growth by inhibiting cell division and elongation (Le Gall et al., 2015). Expansins can allow plant cell walls to loosen. They also participate in biological processes involving plant hormones. ABA promotes radial expansion of cortical cells in the root meristem, resulting in a swollen primary root. Recent transcriptome analysis showed that genes involved in cell wall organization or biogenesis were regulated by ABA, including expansin genes. Induction of expansin genes could rely on the ABA signalling pathway. This finding deepens our understanding of the role of expansin salt stress response. Further investigations will be required to determine if, in this particular case, ABA signalling truly triggers regulation of expansin genes. It would be interesting to estimate quantitatively the expression of the various expansin genes and ABA-signalling factor of this study.

In a recent study on Poncirus trifoliata (Wei et al., 2020) also demonstrated that 4x trifoliate orange (Poncirus trifoliata (L.) Raf.) exhibited enhanced salt tolerance in comparison with 2x counterparts. Similarly to what we found here, theses authors demonstrated that genes related to plant hormone signal transduction were enriched in 4x PO leaves. In addition, genes encoding different antioxidant enzymes were upregulated in 4x genotypes under salt stress. These results tend also corroborates our previous study, showing that 4x genotypes could accumulate higher levels of soluble sugars and proline but less ROS under salt stress compared to their 2x counterparts. A study on transcriptome analysis of salt tolerance of roots in diploid and autotetraploid citrus rootstock (*C. junos* cv. *Ziyang xiangcheng*) (Song et al., 2023) showed that chitin response-related genes were enriched in roots of autotetraploid. The 4x exhibited higher chitinases activity than the diploids under salt stress. These results indicate that in other citrus species, salt stress induced the activation of chitin pathway, thus reducing in ROS accumulation, eventually enhancing salt tolerance of 4x. We found few chitin-responses related genes (**Supplementary Table S2**) in 4x genotypes. It is thus possible that the stress modality applied in our experiment (90 mM NaCl) was not strong enough to effectively observe overexpression of these genes in our experiment. In the future, we would like to test a prolonged and more intense salt stress exposition.

## 5 Conclusion

Tetraploid citrus may hold great potential for rootstock breeding programs and germplasm enhancement, since they harbour better tolerance capacity. The reason underlying 4x’s better tolerance could be ascribed to multiple mechanisms, which makes it difficult to study. Our results demonstrate that stress, ploidy and interaction between ploidy and stress target various gene sets’ responses, leading to a complex adaptation response.

Salt treatment we applied explains the differences obtained between control and stressed genotypes at physiological, biochemical and transcriptomic levels (). Other factors explaining the variability of the results are, in order of importance, genotype and ploidy level. Overall, this work highlights the increasing contrast between samples, depending on genotype and ploidy level, subjected to stress treatment versus control treatment. Our results tend to confirm that genotype and ploidy influence salt stress tolerance in the varieties tested. All genotypes tested showed a downregulation of photosynthesis related processes. Tetraploids genotypes of PO and CL tends to show an enhancement of cell wall remodelling and ion transport related process.

In our previous work, we demonstrated that CL genotypes could have a better adaptive capacity than PO ones, especially the CL4x, even if tetraploidy enhanced tolerance of the sensitive trifoliate orange. If PO is sensitive to salt stress, it is tolerant to cold and CTV. It will be interesting to seek combined parent-tolerance capacity in the PO x CL hybrids and establish whether the 4x hybrids overcome their counterparts’ tolerance ability. In the long term, this knowledge could radically change our vision of plant adaptation, guide breeding programmes and improve tolerance to abiotic stress in citrus fruit.

Finally, it must be stressed that there are still many genes without annotation. Numerous genes are found to code for proteins of unknown function without orthologues in the Arabidopsis genome dataset. Identifying the function of these genes and therefore improving the annotation available for the citrus genome would certainly contribute to a better understanding of CL and PO response to salt stress.

The study of genetic determinants of salt stress tolerance in citrus fruit was approached by means of a large-scale transcriptomic study (RNA-seq). Using this method, our goal was to identify potential variants between CL, which are more resistant to salt stress, and the reference PO which is sensitive to salt stress that could be used to steer the rootstock breeding programs. Moreover, the development of epigenetic approaches on such polyploid plant material, would enable to better understand the genetic regulation mechanisms involved in the heritability traits of adaptation to salt stress.

## Data Availability

The data presented in the study are deposited in the at NCBI repository under the accession PRJNA1020629.

## Footnotes

1 Droc, G., Giraud, D., Belser, C., Labadie, K., Duprat, S., Cruaud, C., et al. (2024). Asuper-pangenome for cultivated citrus reveals evolutive features during the allopatricphase of their reticulate evolution (In press).

## Supplementary material

Supplementary Table S2 GO analysis details and Identification of genes related to theses GO terms in Poncirus trifoliata (PO) and Cleopatra mandarin (CL) 2x and 4x, in control (C) and stressed (S) conditions.
