# Supplementary material for: Comparative transcriptomic analyses of diploid and tetraploid citrus reveal how ploidy level influences salt stress tolerance
Source: Front Plant Sci. 2024 Oct 30;15:1469115. doi: 10.3389/fpls.2024.1469115 (PMC11561191; doi:10.3389/fpls.2024.1469115)
Supplement: Supplementary file 1 [file DataSheet1.pdf]

Supplementary Material

1 Supplementary Data

Article title: Comparative transcriptomic analyses of diploid and tetraploid Citrus reveal how ploidy level could influence salt stress tolerance

Authors: Marie Bonnin[1], Alexandre Soriano[2], Bénédicte Favreau[2], Radia Lourkisti[1], Maëva Miranda[2], Patrick Ollitrault[2], Julie Oustric[1], Liliane Berti[1], Jérémie Santini[1], Raphaël Morillon[2]*

1 Projet Ressources Naturelles Axe Adaptation des végétaux aux changements globaux, UMR CNRS 6134 SPE, Université de Corse, Corsica, France

2 Unité Mixte de Recherche Amélioration Génétique et Adaptation des Plantes méditerranéennes et tropicales (UMR AGAP) Institut, Univ. Montpellier, Centre de coopération Internationale en Recherche Agronomique pour le Développement (CIRAD), Institut National de Recherche pour l'Agriculture, l'Alimentation et l'Environnement (INRAE), Institut Agro, Montpellier, France.

* **Correspondence:** raphael.morillon@cirad.fr **; +334 67 59 37 19.**

The following Supporting Information is available for this article:

Table S1. Plant Material

Table S2 : GO analysis details and Identification of genes related to theses GO terms in Poncirus trifoliata (PO) and Cleopatra mandarin (CL) 2x and 4x, in control (C) and stressed (S) conditions.

Table S3. Specific details regarding the number of reads or gigabytes per sample, the number of clean reads, and the mapping rate

Fig. S1 Discriminant analysis of transcriptomic data under control conditions.

Fig. S2 Venn diagram showing the overlap of differentially expressed genes between 2x and 4x CL (a) leaves and (b) roots and PO (c) leaves and (d) roots.

Fig. S3. GO enrichment map of genes “stress” in CL leaves (A) and roots (B). Nodes represent enriched GO and edges between nodes indicate their functional similarity. Node size is proportional to the number of expressed genes. Intensity of node orange coloration corresponds to adjusted p-value (p-adjust). Edges length between nodes is proportional to the degree of functional similarity

between them. Nodes having very similar biological functions are clustered and labelled with a summarized name.

Fig. S4. GO enrichment map based of ploidy DEGs in PO leaves (A) and roots (B). GO enriched gene sets connected by edges mean that they shared a similarity. Nodes belonging to very similar gene function are clustered and labelled with a summarized name. Node size corresponds to enrichment significance (p-value). Intensity of orange coloration corresponds to adjusted (p-ajust). Node size is proportional to the number of expressed genes. Edges thickness between nodes is proportional to the degree of similarity between them.

Fig. S5. Mapping and count for Cleopatra mandarin.

Fig.S6. Mapping and count for Poncirus trifoliata.

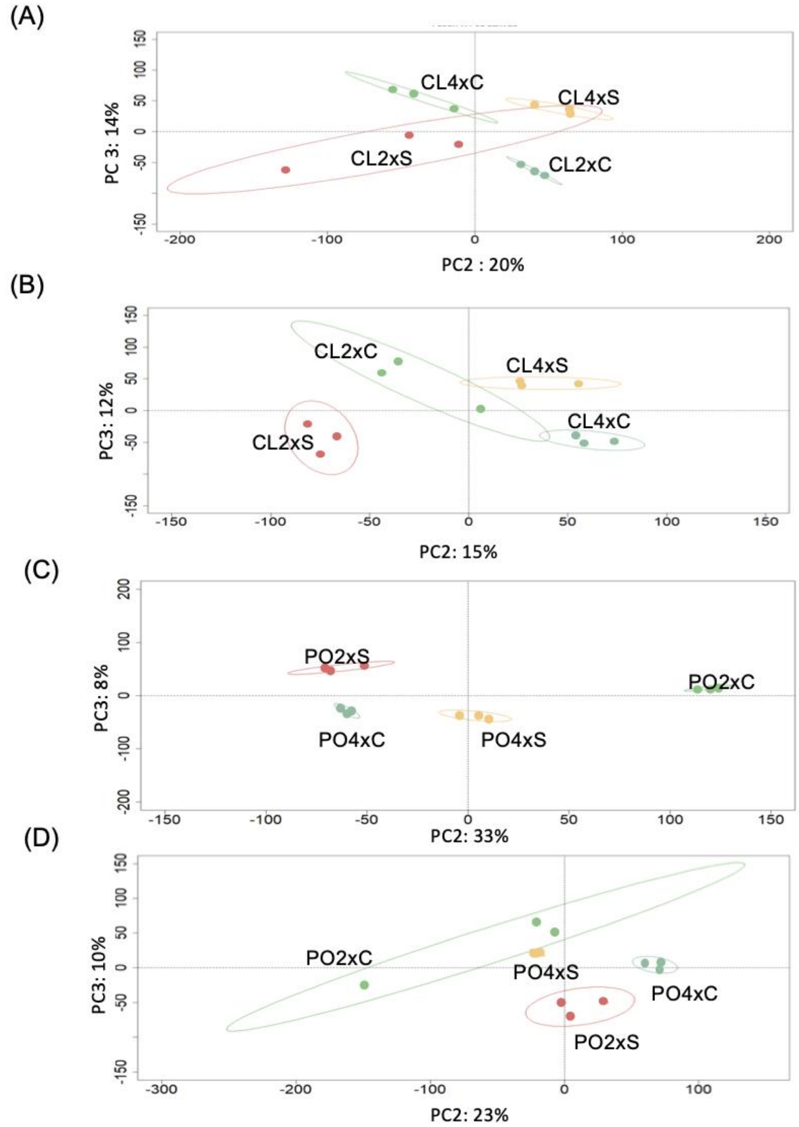

2 Supplementary Figures and Tables

2.1 Supplementary Figures

Fig.S1. Discriminant analysis of transcriptomic data under control conditions. Samples were projected into the space spanned by the second and the third components (PC2 and PC3). PLSDA biplot represented discrimination of Cleopatra mandarin (CL) and Poncirus trifoliata (PO) genotypes at two ploidy levels (2x, 4x) in CL leaves (A), CL roots (B), PO leaves (C) and PO roots (D). Samples are colored according to genotype. Measurements were performed after four weeks of salt stress (90 mM NaCl). Confidence ellipses were set to 95%. C and S represent control and stressed plants, respectively.

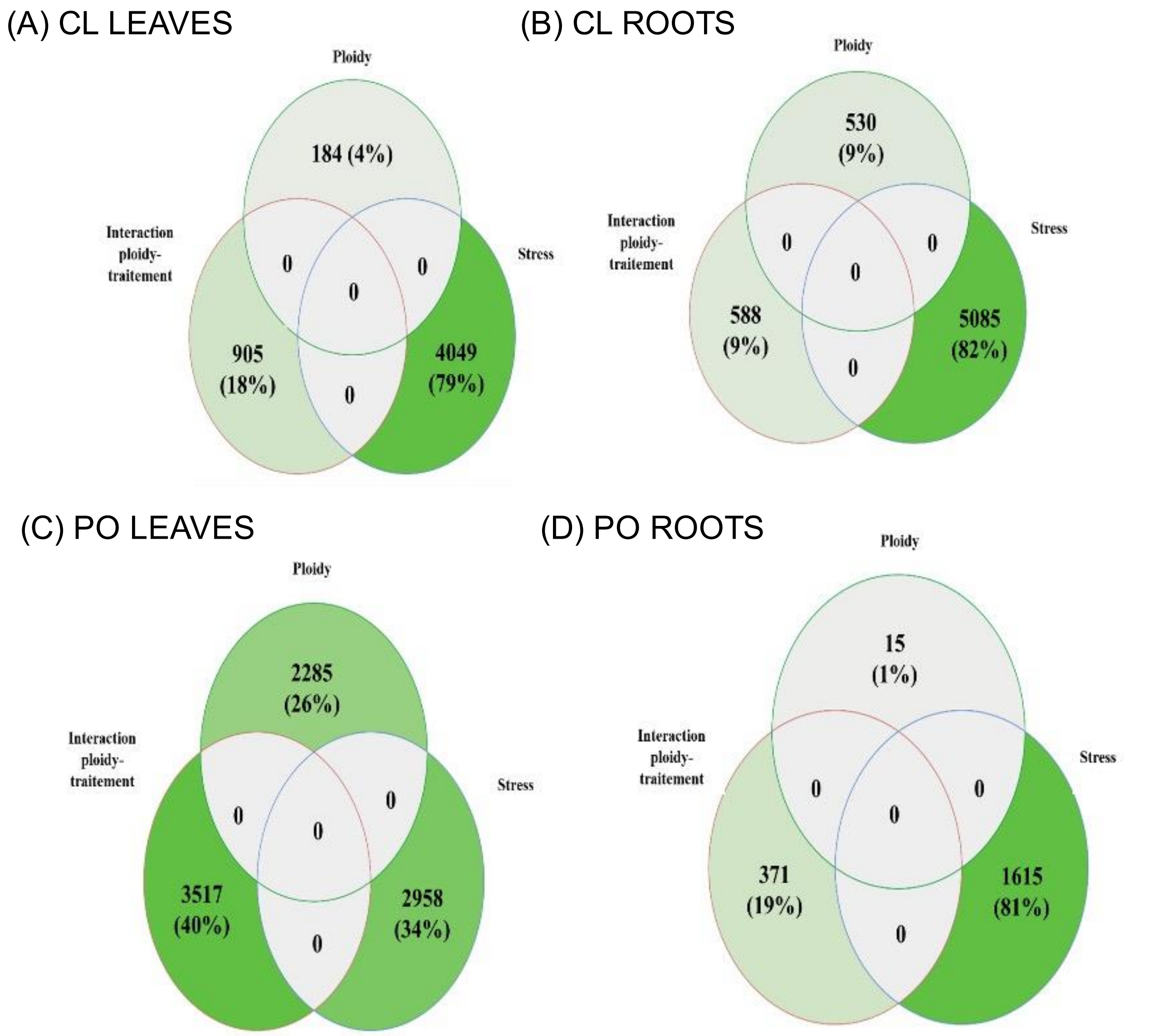

Fig. S2. Venn diagram showing the overlap of differentially expressed genes between 2x and 4x CL (A) leaves and (B) roots and PO (C) leaves and (D) roots. Higher gene count number was represented with deeper color as shown by the legend on graph.

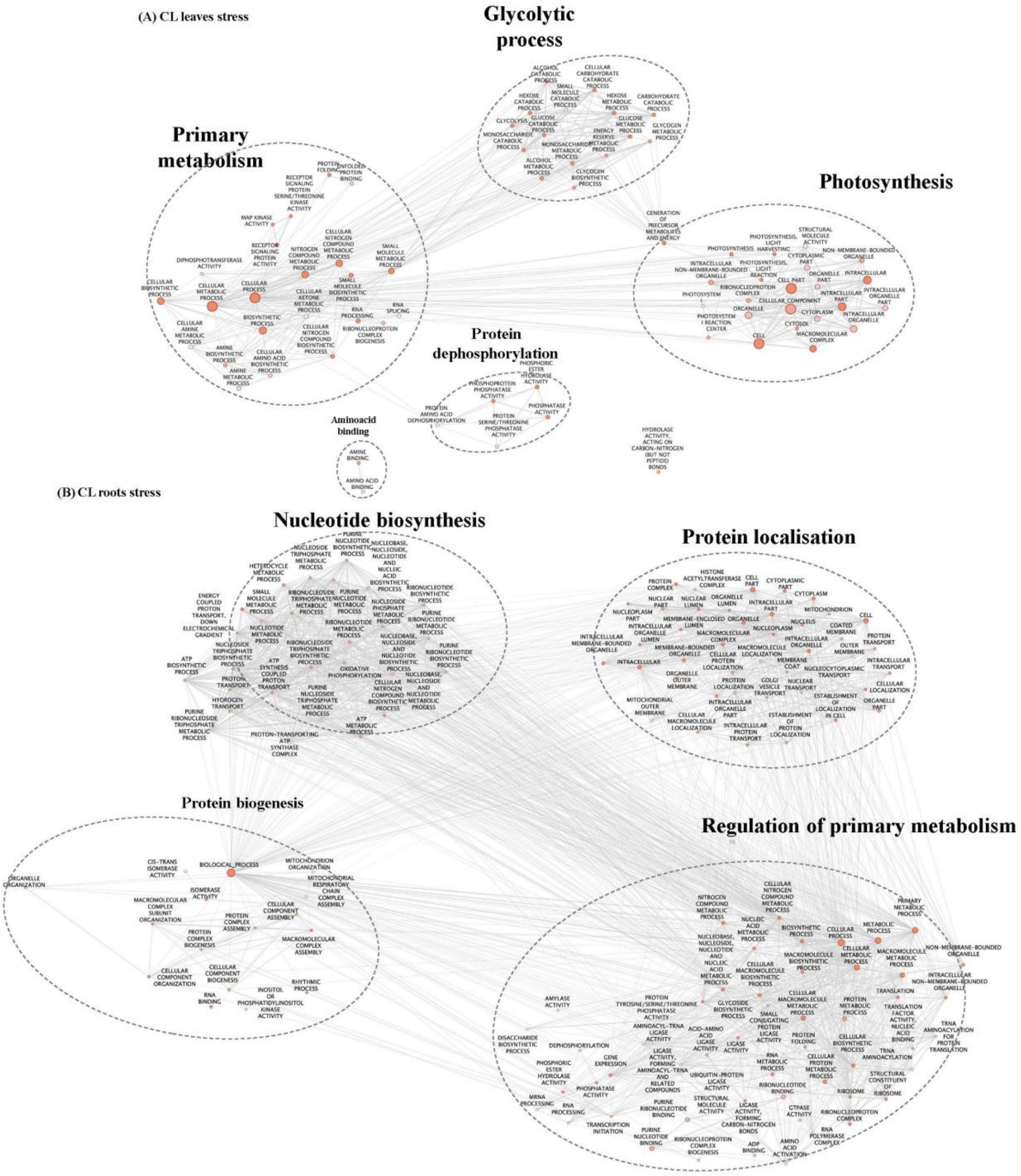
(A) CL leaves stress
Glycolytic process
Primary metabolism
Photosynthesis
Protein dephosphorylation
Aminoacid binding
(B) CL roots stress
Nucleotide biosynthesis
Protein localisation
Protein biogenesis
Regulation of primary metabolism

Fig. S3. GO enrichment map of genes “stress” in CL leaves (A) and roots (B). Nodes represent enriched GO and edges between nodes indicate their functional similarity. Node size is proportional to the number of expressed genes. Intensity of node orange coloration corresponds to adjusted p-value (p-adjust). Edges length between nodes is proportional to the degree of functional similarity between them. Nodes having very similar biological functions are clustered and labelled with a summarized name.

(a) PO leaves ploidy

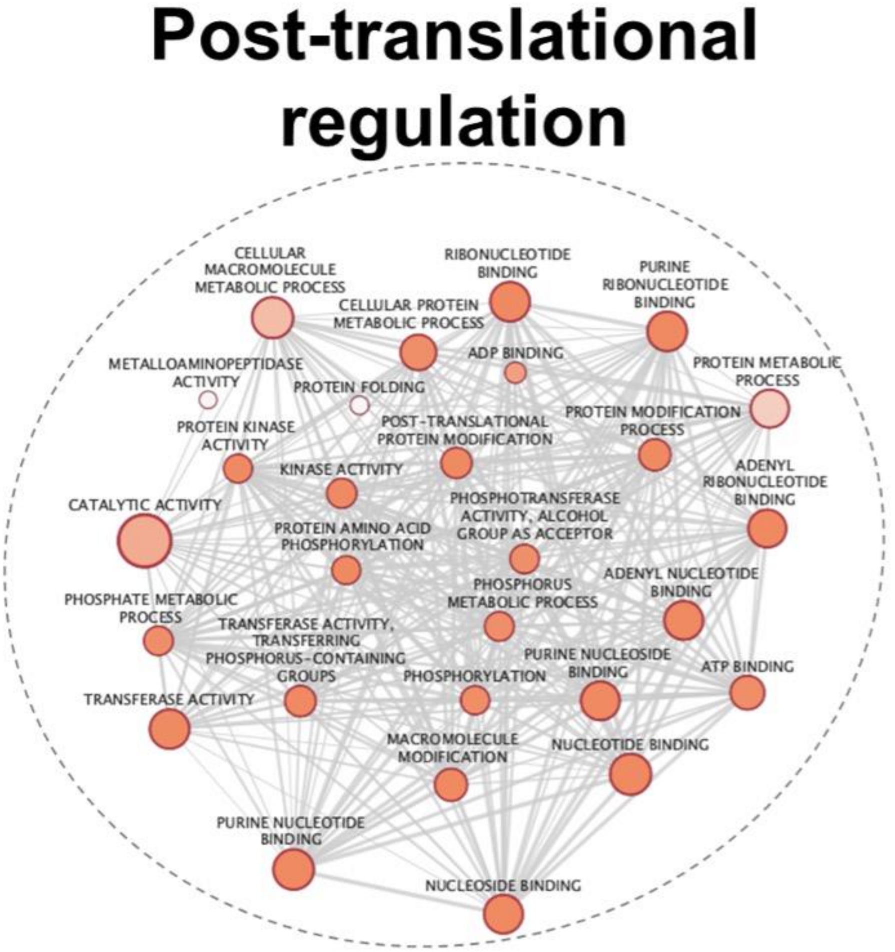

(b) PO roots Ploidy

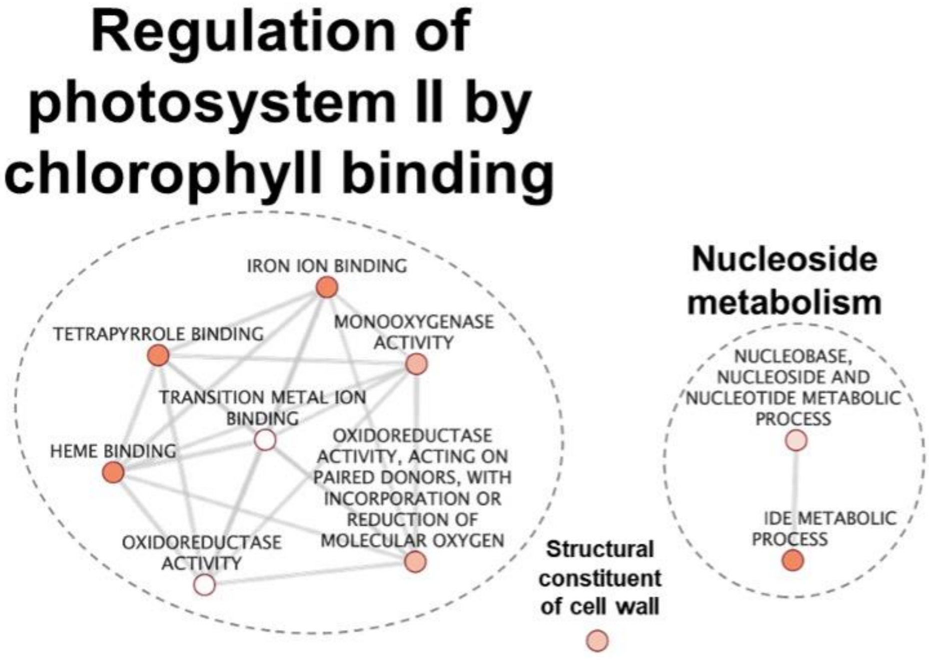

Fig. S4. GO enrichment map based of ploidy DEGs in PO leaves (A) and roots (B). GO enriched gene sets connected by edges mean that they shared a similarity. Nodes belonging to very similar gene function are clustered and labelled with a summarized name. Node size corresponds to enrichment significance (p-value). Intensity of orange coloration corresponds to adjusted (p-ajust). Node size is proportional to the number of expressed genes. Edges thickness between nodes is proportional to the degree of similarity between them.

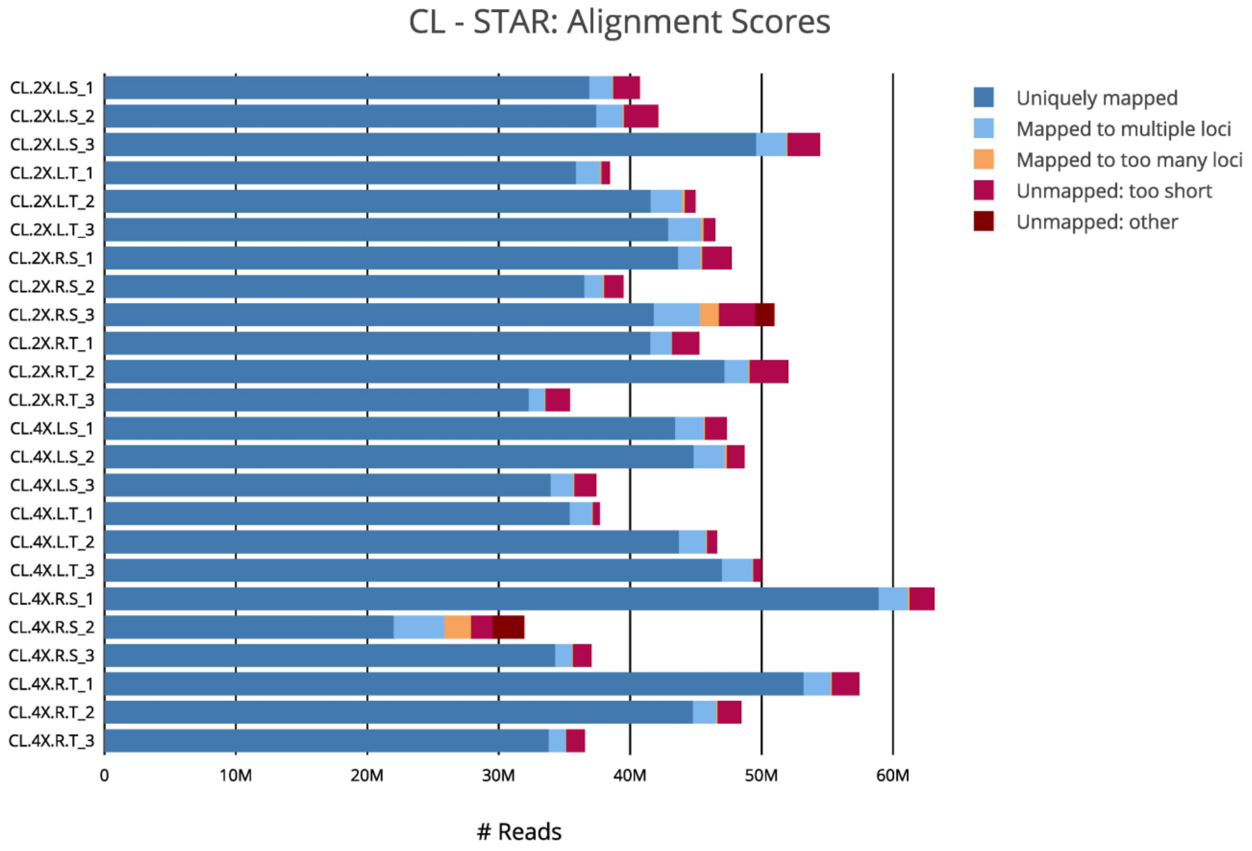
CL - STAR: Alignment Scores
CL.2X.L.S_1
CL.2X.L.S_2
CL.2X.L.S_3
CL.2X.L.T_1
CL.2X.L.T_2
CL.2X.L.T_3
CL.2X.R.S_1
CL.2X.R.S_2
CL.2X.R.S_3
CL.2X.R.T_1
CL.2X.R.T_2
CL.2X.R.T_3
CL.4X.L.S_1
CL.4X.L.S_2
CL.4X.L.S_3
CL.4X.L.T_1
CL.4X.L.T_2
CL.4X.L.T_3
CL.4X.R.S_1
CL.4X.R.S_2
CL.4X.R.S_3
CL.4X.R.T_1
CL.4X.R.T_2
CL.4X.R.T_3
0
10M
20M
30M
40M
50M
60M
Reads
Uniquely mapped
Mapped to multiple loci
Mapped to too many loci
Unmapped: too short
Unmapped: other

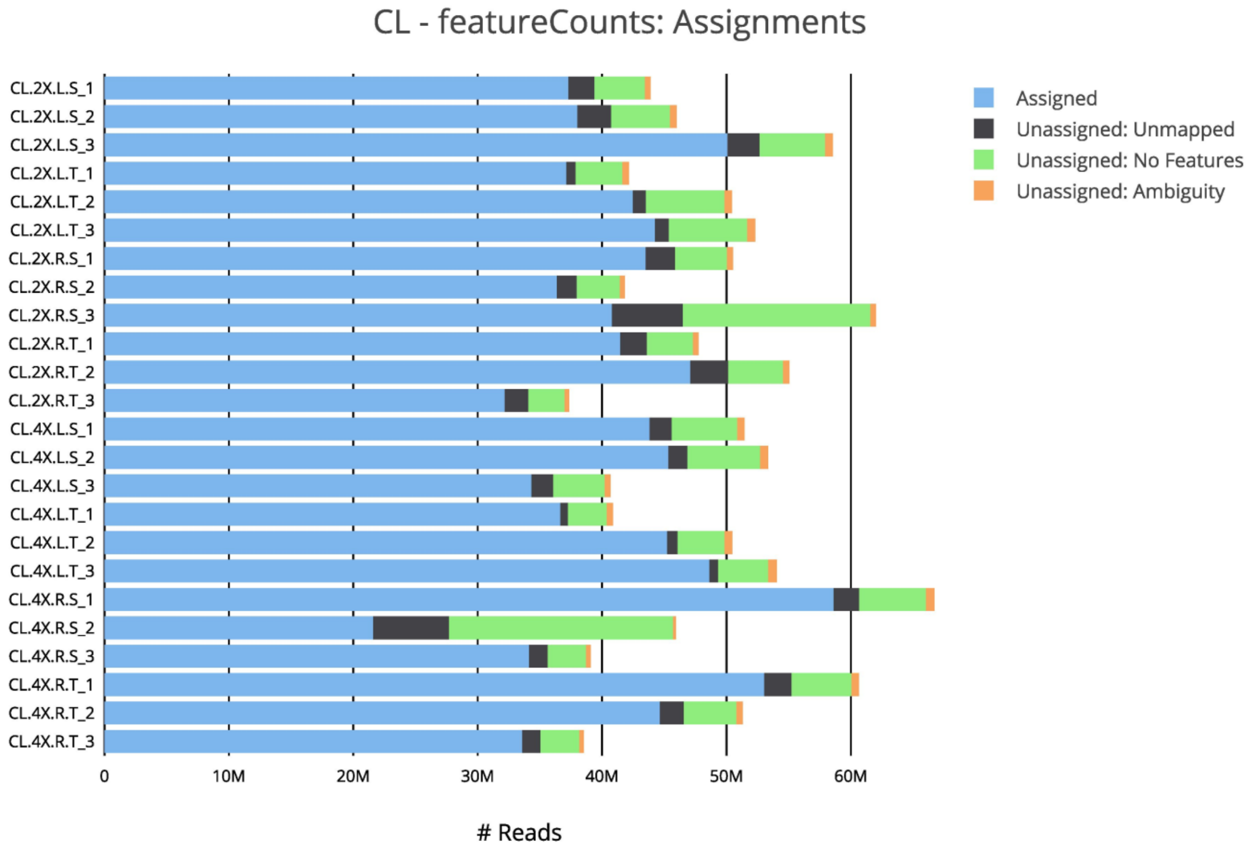
CL - featureCounts: Assignments
CL.2X.L.S_1
CL.2X.L.S_2
CL.2X.L.S_3
CL.2X.L.T_1
CL.2X.L.T_2
CL.2X.L.T_3
CL.2X.R.S_1
CL.2X.R.S_2
CL.2X.R.S_3
CL.2X.R.T_1
CL.2X.R.T_2
CL.2X.R.T_3
CL.4X.L.S_1
CL.4X.L.S_2
CL.4X.L.S_3
CL.4X.L.T_1
CL.4X.L.T_2
CL.4X.L.T_3
CL.4X.R.S_1
CL.4X.R.S_2
CL.4X.R.S_3
CL.4X.R.T_1
CL.4X.R.T_2
CL.4X.R.T_3
0
10M
20M
30M
40M
50M
60M
Reads
Assigned
Unassigned: Unmapped
Unassigned: No Features
Unassigned: Ambiguity

Fig. S5. Mapping and count for Cleopatra mandarin.

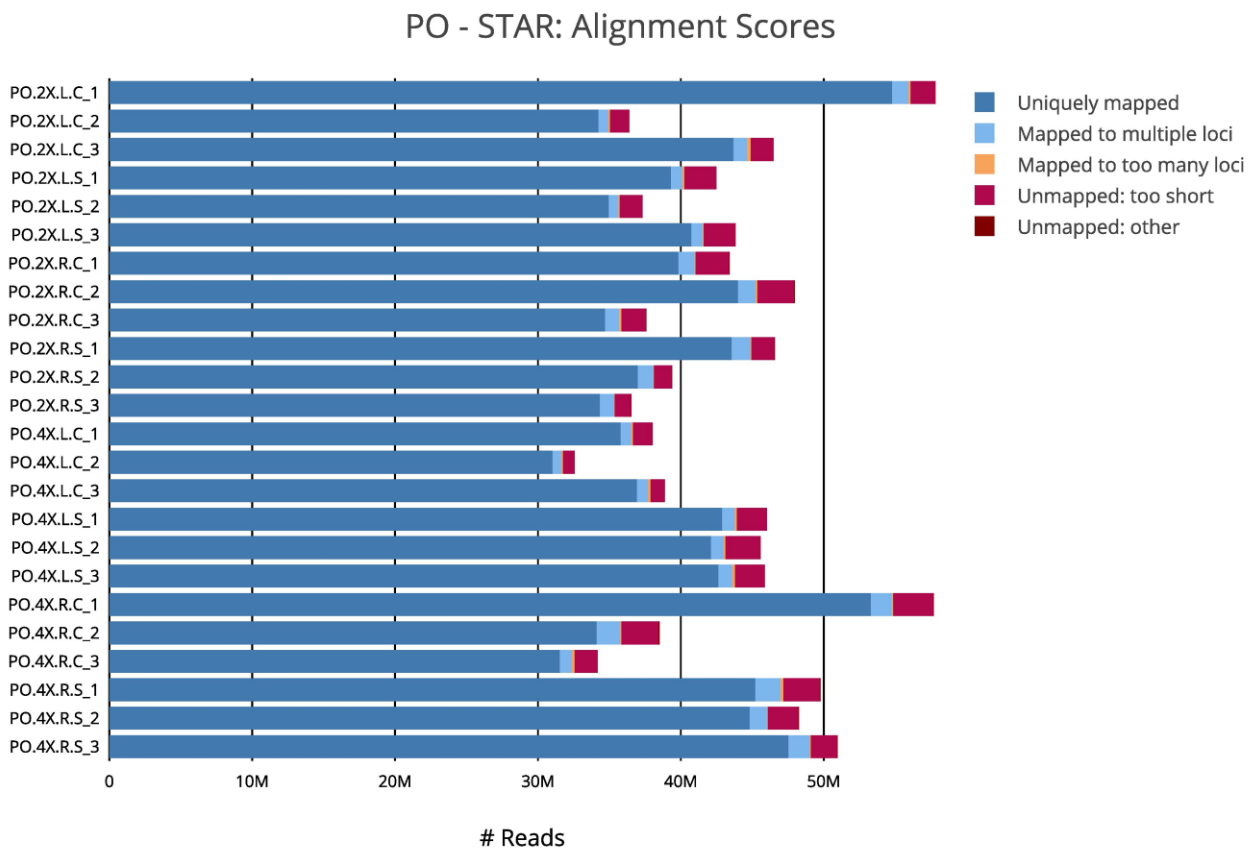
PO - STAR: Alignment Scores
PO.2X.L.C_1
PO.2X.L.C_2
PO.2X.L.C_3
PO.2X.L.S_1
PO.2X.L.S_2
PO.2X.L.S_3
PO.2X.R.C_1
PO.2X.R.C_2
PO.2X.R.C_3
PO.2X.R.S_1
PO.2X.R.S_2
PO.2X.R.S_3
PO.4X.L.C_1
PO.4X.L.C_2
PO.4X.L.C_3
PO.4X.L.S_1
PO.4X.L.S_2
PO.4X.L.S_3
PO.4X.R.C_1
PO.4X.R.C_2
PO.4X.R.C_3
PO.4X.R.S_1
PO.4X.R.S_2
PO.4X.R.S_3
0
10M
20M
30M
40M
50M
Reads
Uniquely mapped
Mapped to multiple loci
Mapped to too many loci
Unmapped: too short
Unmapped: other

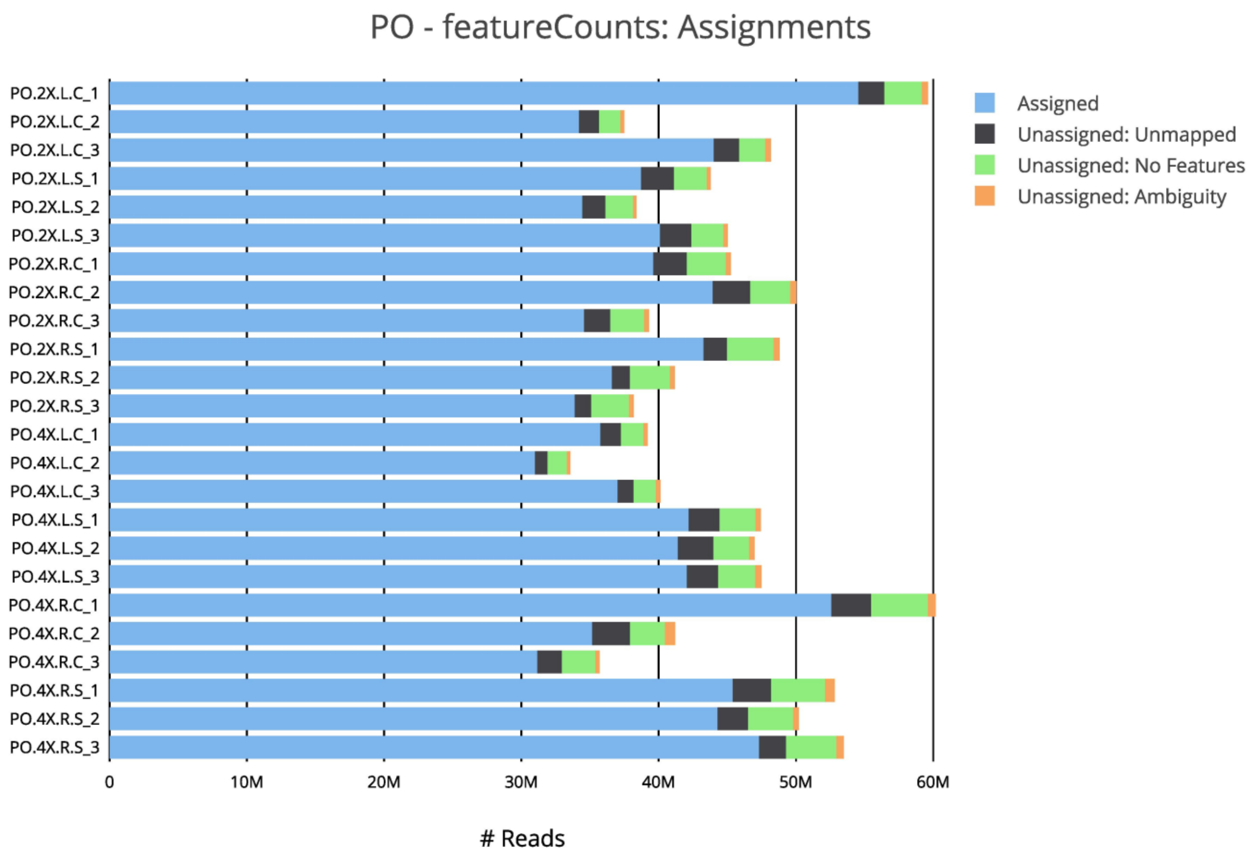
PO - featureCounts: Assignments
PO.2X.L.C_1
PO.2X.L.C_2
PO.2X.L.C_3
PO.2X.L.S_1
PO.2X.L.S_2
PO.2X.L.S_3
PO.2X.R.C_1
PO.2X.R.C_2
PO.2X.R.C_3
PO.2X.R.S_1
PO.2X.R.S_2
PO.2X.R.S_3
PO.4X.L.C_1
PO.4X.L.C_2
PO.4X.L.C_3
PO.4X.L.S_1
PO.4X.L.S_2
PO.4X.L.S_3
PO.4X.R.C_1
PO.4X.R.C_2
PO.4X.R.C_3
PO.4X.R.S_1
PO.4X.R.S_2
PO.4X.R.S_3
0
10M
20M
30M
40M
50M
60M
Reads
Assigned
Unassigned: Unmapped
Unassigned: No Features
Unassigned: Ambiguity

Fig.S6. Mapping and count for Poncirus trifoliata.

2.2 Supplementary Table

Table S1. Plant Material

**Common name**	**Scientific name**	**Ploidy level**	**Tissues samples**	**Number of biological replicates**	**ICVN***
Cleopatra mandarin	*C. reshni*	2x	Roots<br>leaves	3	ICVN-0110274
Trifoliate orange	*P. trifoliata*	2x	Roots<br>leaves	3	ICVN-0110081
Cleopatra mandarin	*C. reshni*	4x	Roots<br>leaves	3	ICVN-0101110
Trifoliate orange	*P. trifoliata*	4x	Roots<br>leaves	3	ICVN-01011106

*The first 01 in the ICVN number stands for "variety from the San Giuliano collection"; other collections around the world have a different number. The next number, always 1 or 0, means "micrografted, without pathogens= 0"; "seedling not micrografted = 1". The following numbers are the registration number in the collection.

Table S2: GO analysis details and Identification of genes related to theses GO terms in Poncirus trifoliata (PO) and Cleopatra mandarin (CL) 2x and 4x, in control (C) and stressed (S) conditions.

Table S2 has been uploaded separately in xlsx format.

Table S3. Specific details regarding the number of reads or gigabytes per sample, the number of clean reads, and the mapping rate
