# Supplementary material for: Comparative transcriptomic analyses of diploid and tetraploid citrus reveal how ploidy level influences salt stress tolerance
Source: Front Plant Sci. 2024 Oct 30;15:1469115. doi: 10.3389/fpls.2024.1469115 (PMC11561191; doi:10.3389/fpls.2024.1469115)
Supplement: Supplementary file 2 [file Table1.docx]

## Supplementary Table

**Table S1.** Plant Material

| **Common name** | **Scientific name** | **Ploidy level** | **Tissues samples** | **Number of biological replicates** | **ICVN*** |
| --- | --- | --- | --- | --- | --- |
| *Cleopatra mandarin* | *C. reshni* | 2x | Roots  leaves | 3 | ICVN-0110274 |
| *Trifoliate orange* | *P. trifoliata* | 2x | Roots  leaves | 3 | ICVN-0110081 |
| *Cleopatra mandarin* | *C. reshni* | 4x | Roots  leaves | 3 | ICVN-0101110 |
| *Trifoliate orange* | *P. trifoliata* | 4x | Roots  leaves | 3 | ICVN-01011106 |

*The first 01 in the ICVN number stands for "variety from the San Giuliano collection"; other collections around the world have a different number. The next number, always 1 or 0, means "micrografted, without pathogens= 0"; "seedling not micrografted = 1". The following numbers are the registration number in the collection.
